# Investigating the impact of environmental enrichment on proteome and neurotransmitter‐related profiles in an animal model of Alzheimer's disease

**DOI:** 10.1111/acel.14231

**Published:** 2024-07-01

**Authors:** Yunkwon Nam, Sujin Kim, Yong Ho Park, Byeong‐Hyeon Kim, Soo Jung Shin, Seol Hwa Leem, Hyun Ha Park, Gukhwa Jung, Jeongbeen Lee, Hyung‐Gun Kim, Doo‐Han Yoo, Hak Su Kim, Minho Moon

**Affiliations:** ^1^ Department of Biochemistry, College of Medicine Konyang University Daejeon South Korea; ^2^ Research Institute for Dementia Science Konyang University Daejeon South Korea; ^3^ NeuroVis Inc. Cheonan South Korea; ^4^ Department of Occupational Therapy Konyang University Daejeon South Korea; ^5^ Veterans Medical Research Institute Veterans Health Service Medical Center Seoul South Korea

**Keywords:** 5XFAD mice, Alzheimer's disease, environmental enrichment, neurotransmitter, proteomics

## Abstract

Alzheimer's disease (AD) is a neurodegenerative disorder associated with behavioral and cognitive impairments. Unfortunately, the drugs the Food and Drug Administration currently approved for AD have shown low effectiveness in delaying the progression of the disease. The focus has shifted to non‐pharmacological interventions (NPIs) because of the challenges associated with pharmacological treatments for AD. One such intervention is environmental enrichment (EE), which has been reported to restore cognitive decline associated with AD effectively. However, the therapeutic mechanisms by which EE improves symptoms associated with AD remain unclear. Therefore, this study aimed to reveal the mechanisms underlying the alleviating effects of EE on AD symptoms using histological, proteomic, and neurotransmitter‐related analyses. Wild‐type (WT) and 5XFAD mice were maintained in standard housing or EE conditions for 4 weeks. First, we confirmed the mitigating effects of EE on cognitive impairment in an AD animal model. Then, histological analysis revealed that EE reduced Aβ accumulation, neuroinflammation, neuronal death, and synaptic loss in the AD brain. Moreover, proteomic analysis by liquid chromatography–tandem mass spectrometry showed that EE enhanced synapse‐ and neurotransmitter‐related networks and upregulated synapse‐ and neurotransmitter‐related proteins in the AD brain. Furthermore, neurotransmitter‐related analyses showed an increase in acetylcholine and serotonin concentrations as well as a decrease in polyamine concentration in the frontal cortex and hippocampus of 5XFAD mice raised under EE conditions. Our findings demonstrate that EE restores cognitive impairment by alleviating AD pathology and regulating synapse‐related proteins and neurotransmitters. Our study provided neurological evidence for the application of NPIs in treating AD.

## Graphical abstract

Graphical illustration of the therapeutic mechanisms of EE on cognitive deficit in AD. Graphical illustration of the therapeutic mechanism of EE on cognitive deficit in AD. The therapeutic mechanisms of EE are (1) attenuation of Aβ accumulation, neuroinflammation, synaptic loss, and neuronal loss, (2) upregulation of the synapse‐ and neurotransmitter‐related proteome, and (3) regulation of neurotransmitter concentrations in the AD brain. Consequently, EE intervention alleviates cognitive dysfunction in animal models of AD.

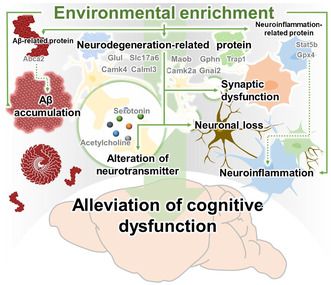

## 1 INTRODUCTION

More than 55 million people and their families worldwide have been diagnosed with dementia (“^2020^ Alzheimer's disease facts and figures,”, 2020). Alzheimer's disease (AD) is the most common type of dementia characterized by progressive impairment of behavioral and cognitive functions. The pathological characteristics of AD are the accumulation of amyloid beta (Aβ) and neurofibrillary tangle (NFT) composed of hyperphosphorylated tau (Long & Holtzman, 2019). Numerous pharmacological research has been performed to search for helpful AD treatments, including disease‐modifying therapies targeting Aβ and tau, as well as symptomatic therapies aiming to improve cognitive, neuropsychiatric, and behavioral symptoms. Despite the development of disease‐modifying therapies such as aducanumab (brand name: Aduhelm) (Budd Haeberlein et al., 2022) and lecanemab (brand name: Leqembi) (Swanson et al., 2021; van Dyck et al., 2022), unfortunately, these treatments do not seem to delay or reverse the progression of AD significantly. In contrast, symptomatic treatments such as donepezil (brand name: Aricept), galantamine (brand name: Radadyne), and rivastigmine (brand name: Exelon) show greater promise in providing short‐term cognitive improvement for the treatment of AD. Nonetheless, acetylcholinesterase inhibitors, which remain the primary pharmacological agents for alleviating AD symptoms, have limited effects, and increasing the dosage or duration of treatment does not result in a dramatic cure for AD (Bohnen et al., 2005; Homma et al., 2016).

The difficulty in developing pharmacological treatments for AD has aroused increasing interest in developing alternative non‐pharmacological interventions (NPIs). Increasing evidence shows that NPIs may represent an optimal approach for alleviating physical and mental symptoms without inducing severe side effects (Olazaran et al., 2010; Scales et al., 2018; Zucchella et al., 2018). Moreover, NPIs can provide personalized treatment approaches based on the individual conditions of patients with AD (Sagud et al., 2021). Furthermore, NPIs can enhance cognitive function and help maintain activities of daily living (ADL) in patients with AD (Sharew, 2022). NPIs, including cognitive intervention, occupational therapy (OT), aromatherapy, music therapy, and psychological therapy, effectively alleviate the symptoms of neurodegenerative diseases, including AD (Smallfield & Heckenlaible, 2017; Zucchella et al., 2018). Recently, programs of NPIs based on epidemiological discovery have emerged, such as exercise in adults with mild memory problems (EXERT; NCT number: NCT02814526) and the Finnish geriatric intervention study to prevent cognitive impairment and disability (FINGER; NCT number: NCT 01041989), and have shown a beneficial effect on various domains of cognitive function in individuals at risk of cognitive decline (Kivipelto et al., 2013; Rosenberg et al., 2018). In particular, our meta‐analysis reported that OT with a cognition‐oriented approach has beneficial effects on cognitive dysfunction and ADL impairment in patients with AD (Ham et al., 2021). Moreover, sensory stimulation‐based NPIs have been reported to alleviate the behavioral and psychological symptoms of dementia (BPSD) significantly and cognitive dysfunction in patients with AD (Kim et al., 2012; Yang et al., 2021).

Environmental enrichment (EE) provides greater sensory, physical, cognitive, and social stimulation and is a representative experimental intervention for NPIs (Figuracion & Lewis, 2021). Interestingly, EE triggers a cascade of anatomical and molecular changes within the AD brain and contributes to ameliorating in symptoms of AD (Herring et al., 2009; Lahiani‐Cohen et al., 2011; Lazarov et al., 2005). EE promotes the degradation of Aβ through the increase of neprilysin activity in AD animal models (Lazarov et al., 2005). Moreover, EE decreased NFT in the hippocampus and cerebral cortex and alleviated cognitive decline in a tauopathy mouse model (Lahiani‐Cohen et al., 2011). One study reported that EE reduced microgliosis and restored impaired adult hippocampal neurogenesis (AHN) in an animal model of AD (Herring et al., 2009). However, the molecular mechanisms underlying the EE‐induced anatomical and molecular changes in AD remain unknown.

Several studies have revealed that EE ameliorates the symptoms of patients with AD and AD animal models (Herring et al., 2009; Lahiani‐Cohen et al., 2011; Lazarov et al., 2005; Yang et al., 2021). Although considerable progress has been achieved in exploring the beneficial effects of EE on AD, our understanding of the therapeutic mechanisms of EE in AD is limited. This study aimed to elucidate the therapeutic mechanisms by which EE alleviates cognitive deficits in AD. We confirmed the effects of EE on cognitive dysfunction in 5XFAD mice. Next, we investigated the therapeutic mechanisms of EE using histological, proteomic, and neurotransmitter‐related analyses in an animal model of AD. Our results may provide dementia clinicians with neurological evidence to support the use of NPIs, including EE intervention.

## 2 MATERIALS AND METHODS

### 2.1 Animals

**FIGURE 1:**
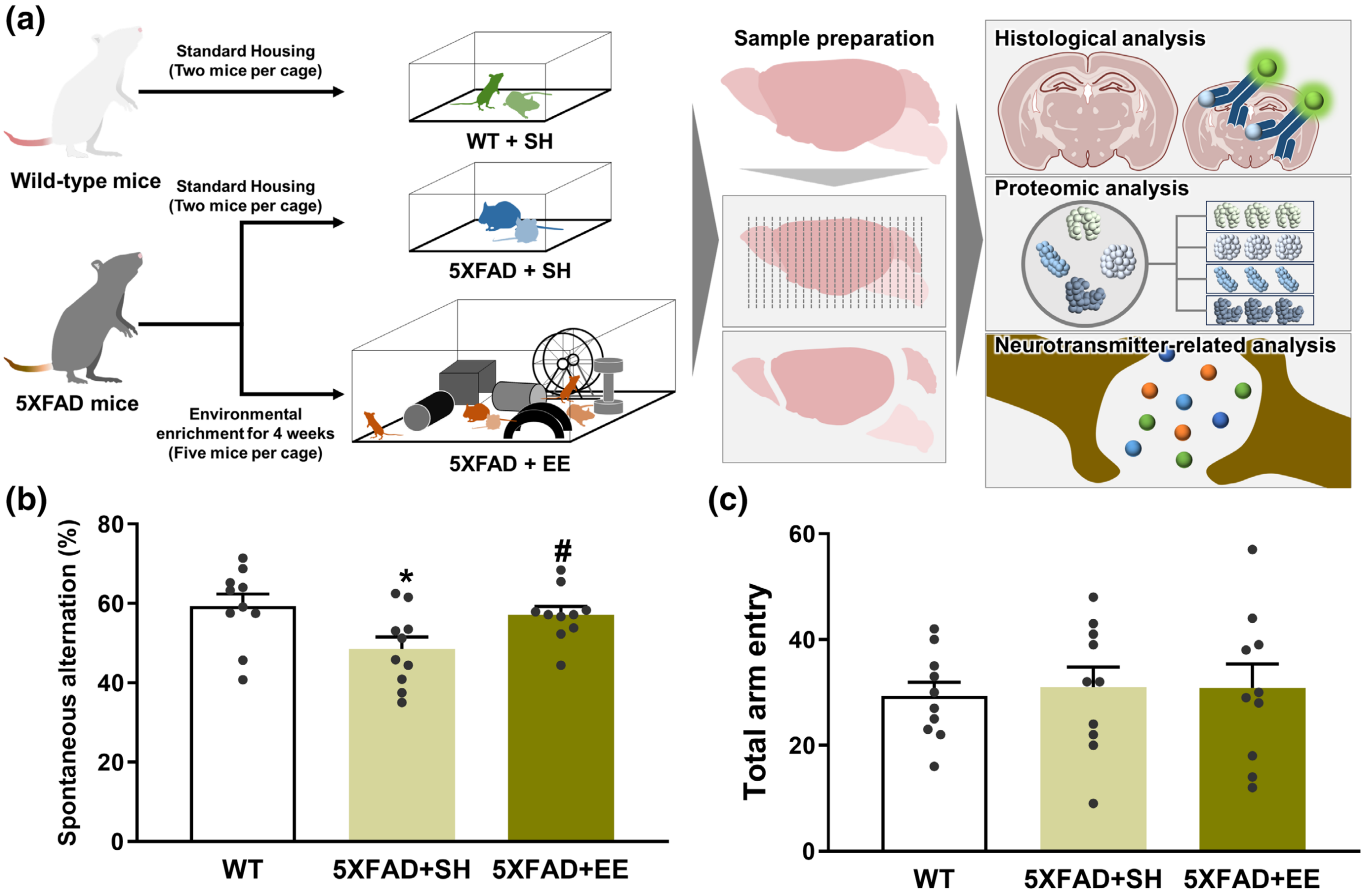
Effect of environmental enrichment (EE) intervention on cognitive impairment of an animal model of Alzheimer’s disease (AD). (a) Schematic workflow of histological, proteomic, and neurotransmitter‐related analysis to examine the mechanisms underlying the therapeutic effect of EE on AD. (b) Spontaneous alteration and (c) total arm entry during 8‐min sessions were measured. Values are expressed as the mean ± standard error of the mean (SEM) (*n* = 10 in standard housing (SH)‐exposed WT mice; *n* = 10 in SH‐exposed 5XFAD mice; and *n* = 10 in EE‐exposed 5XFAD mice). Statistical analysis between three groups was evaluated by one‐way ANOVA, followed by Fisher's LSD test. *p < 0.05 shows significant differences compared with the SH‐exposed WT mice. ^#^ *p* < 0.05 shows significant differences compared with the SH‐exposed 5XFAD mice.

The 5XFAD mice (Tg6799 Stock #006554; Jackson Laboratory, Bar Harbor, Maine, USA) have the following five familial AD mutations: the human presenilin‐1 (*PSEN1*) transgenes bearing M146 and L286 mutations, and human *APP* transgenes bearing Swedish (K607 N and M671L), London (V717I) and Florida (I716V) mutations. Genotyping was conducted using polymerase chain reaction (PCR) of tail DNA, which can reflect the genotype of an individual, to distinguish between 5XFAD and wild‐type (WT). Three groups of female 5XFAD and WT mice at the age of 8 months were used for behavioral tests, histological analysis, and proteomic analysis: (1) WT mice + standard housing (SH; *n* = 10), (2) 5XFAD mice + SH (*n* = 10), and (3) 5XFAD mice + EE (*n* = 10). The brains of five mice from each group were subjected to proteomic and histological analyses. For neurotransmitter‐related analyses, female 5XFAD and WT mice at the age of 8 months were categorized into three groups: (1) WT mice + SH (*n* = 6), (2) 5XFAD mice + SH (*n* = 6), and (3) 5XFAD mice + EE (*n* = 5) (Figure 1a). The care and maintenance of mice in this study followed the protocols approved by the Institutional Animal Care and Use Committee of Konyang University. This animal study was approved by the Ethics Committee of Konyang University (project identification code: P‐22‐11‐E‐01).

### 2.2 Study design and EE condition

Through preliminary research, we conducted an EE experiment by selecting the period when changes in AHN were most evident following the EE intervention. Changes in AHN were most pronounced at week 4 following the EE intervention (Figure S1). Eight‐month‐old 5XFAD mice were randomly distributed into two experimental groups that were exposed to SH or EE for 4 weeks. Age‐matched WT littermate mice were housed under SH conditions for 4 weeks and were used as the control group. WT mice were bred under SH conditions and were used as the control group. The SH group used a standard laboratory mouse cage with dimensions of 30 cm length, 20 cm width, and 15 cm height, whereas the EE group used larger cages measuring 43.5 cm length, 29 cm width, and 15 cm height, respectively. A running wheel, tunnel, mouse igloo with an angled running track, stairs, and seesaw were placed inside the EE cage, which was replaced or rearranged every week to create a sense of novelty, and the food location was changed every time (Figure S2). Water and food were provided ad libitum. In addition, mice in the SH group were housed with two mice per cage, whereas mice in the EE group were housed with five mice per cage to maintain social interactions.

### 2.3 Behavior test

As previously described, short‐term spatial memory was examined using the Y‐maze (Shin et al., 2021). The environment for the behavioral experiment was composed of a temperature of 23 ± 1°C and a humidity of 60 ± 10%. Each arm of the Y‐maze with three arms forms an equal angle of 120° with a length of 30 cm × width of 5 cm × height of 15 cm and is made of black acrylic. One week before the Y‐maze task, preexperimental habituation was conducted to allow the mice to acclimate to the experimental environment, which was placed in an environment similar to the behavioral test and handled for 5 min every day. After handling the mice, they were placed inside the Y‐maze for 30 s to suppress their fear of unfamiliar environments. As mice become accustomed to unfamiliar environments through habituation, atypical responses such as anxiety, and freezing are prevented. Mice were positioned in the middle of the Y‐maze and allowed to explore each arm after the final day of EE exposure. Throughout the 8‐min experiment, the number of arm entries per mouse and the total number of spontaneous alternations were manually counted. Locomotor activity was measured based on the overall number of arm entries. Spontaneous alternation was defined as consecutive non‐repeated entries into all arms (such as ABC, BCA, or CAB). [(the number of alterations)/(the total number of arms entered‐2)] × 100 was used to calculate the percentage of alterations.

### 2.4 Preparation of brain tissue

After the behavioral experiment, animals were anesthetized using a 250 μg/kg intraperitoneal injection of Avertin (Tribromoethanol; Sigma‐Aldrich, St. Louis, MO, USA). The animals were transcardially infused with 0.05 M phosphate‐buffered saline (PBS) and then fixed in a 0.1 M phosphate buffer (PB) containing cold 4% paraformaldehyde (PFA). After the brain tissue was removed, the brain tissues were fixed in a 4% PFA solution for 20 h at 4°C and immersed in a cryoprotectant solution made of 30% sucrose in PBS. The tissues were sliced into 30‐μm‐thick coronal planes at 25°C using a cryostat (Leica Biosystems, Wetzlar, Germany) and then preserved at 4°C in a cryoprotectant solution (0.05 M PB with 25% ethylene glycol and 25% glycerol) to prepare the tissues for immunohistochemistry.

### 2.5 Immunohistochemistry

To examine immunoreactivity, four to six brain slices were collected at intervals of 270–390 μm in the region between −2.70 and −3.80 mm / +2.58 and +3.20 from the bregma. (Figure S3). To examine the effect of EE on alteration of AHN in C57BL/6 mice, two to three brain slices were collected at intervals of 150–240 μm between −1.70 and −2.18 mm from bregma (Figure S3). The free‐floating sections were shortly washed in PBS before being incubated with one of the primary antibodies listed below overnight at 4°C: mouse anti‐neuronal nuclear antigen (NeuN) antibody (1:100; Merck KGaA, Darmstadt, Germany) mouse anti‐4G8 antibody (1:2000; BioLegend, San Diego, CA, USA), goat anti‐ionized calcium‐binding adapter molecule 1 (Iba‐1) antibody (1:1000; Abcam, Cambridge, MA, USA), rat anti‐glia fibrillary acidic protein (GFAP) antibody (1:500; Thermo Fisher Scientific Inc.), mouse anti‐synaptophysin (SYN) antibody (1:500; Sigma‐Aldrich), goat anti‐doublecortin (DCX) antibody (1:1000; Santa Cruz Biotechnology, Dallas, TX, USA) and rabbit anti‐Ki67 antibody (1:2000; Abcam). Each of these antibodies was prepared at a specific dilution ratio in PBS containing 0.5 mg/mL bovine serum albumin and 0.3% Triton X‐100. For antigen retrieval, the brain tissue was treated with 70% formic acid for 20 min before being incubated with the anti‐4G8 antibody. Sections were then incubated for 50 min at room temperature with the following secondary antibodies: donkey Alexa 488‐conjugated anti‐mouse IgG, donkey Alexa 594‐conjugated anti‐mouse IgG, donkey Alexa 594‐conjugated anti‐goat IgG, and donkey Alexa 488‐conjugated anti‐rat IgG (1:300, Thermo Fisher Scientific) in PBS contained 0.3% Triton X‐100. Fluoroshield mounting medium containing 4,6‐diamidino‐2‐phenylindole (DAPI; Sigma‐Aldrich) was used to mount the immunostained tissues on slides and coverslips.

### 2.6 Image acquisition and analysis

Images were captured using a Zeiss LSM 700 microscope (Carl Zeiss AG, Oberkochen, Germany) and analyzed using ImageJ software (National Institutes of Health, Bethesda, MD, USA) to quantify immunoreactivity. Histological quantification, statistical analyses, and image acquisition were performed in a blinded manner. We quantified the area percentages of 4G8 immune‐positive signals in the subiculum and frontal cortex. The Iba‐1 and GFAP positive signals were quantified as area fractions (%) in the subiculum. NeuN‐positive cells were calculated as the number of NeuN‐positive cells/mm^2^ in the subiculum and frontal cortex. SYN immunoreactivity was quantified as fluorescence intensity (optical density) in the subiculum and frontal cortex. DCX and Ki‐67 immunoreactivity were measured as the number of positive cells/lengths of the DG.

### 2.7 Protein extraction

After the behavioral test, the mice received an intraperitoneal injection of a 250 μg/kg Avertin dose to induce anesthesia. Each frontal cortex extracted from mice was dissolved in 100 μL of 5% SDS sample buffer. Subsequently, each sample was treated with 20 mM dithiothreitol in 50 mM ammonium bicarbonate, reduced for 10 min at 95°C, and then alkylated with 40 mM iodoacetamide in 50 mM ammonium bicarbonate for 30 min in darkness. S‐TRAP™ (Protifi, Farmingdale, NY, USA) was used to prepare proteomic samples rapidly and consistently. Denatured but not digested proteins were bound to S‐TRAP™. Each sample was incubated with 12.5 μg of sequencing‐grade modified trypsin/LysC (Promega, Madison, Wisconsin, USA) in a 50 mM ammonium bicarbonate solution (pH 7.8) using an S‐TRAP column overnight at 37°C. A portion of the eluted peptides was dried and analyzed. The samples were resuspended in 0.1% formic acid before drying for liquid chromatography–tandem mass spectrometry (LC–MS) analysis.

### 2.8 Nano‐LC‐ESI‐MS/MS analysis

The UltiMate™ 3000 RSLCnano system, which is connected to a Q Exactive™ Plus Hybrid Quadrupole‐Orbitrap™ mass spectrometer with a nano‐electrospray ionization source, was used to examine the peptide samples (Thermo Fisher Scientific, Waltham, MA, USA). Tryptic peptides from the bead column were reconstituted in 100 μL of 0.1% FA in water and separated over 200 min (250 nL/min) using a 5%–40% acetonitrile gradient in 0.1% FA and 5% DMSO for 150 min at 50°C on an Acclaim™ Pepmap 100 C18 column (500 mm × 75 μm i.d., 3 μm, 100 Å; Thermo Fisher Scientific) fitted with a C18 Pepmap trap column (20 mm × 100 μm i.d., 5 μm, 100 Å; Thermo Fisher Scientific). Mass spectra were obtained in the data‐dependent mode with an automatic changeover between the full scan and the top 20 data‐dependent MS/MS scans. The goal value for the full‐scan MS spectra was a 3,000,000 AGS target with a maximum injection duration of 100 ms and a resolution of 70,000 chosen from a 350 to 1800 *m*/*z* range.

### 2.9 Data search, statistical analysis, and bioinformatic analysis

The SwissProt human database and Sequest HT on Proteome Discoverer (Version 2.2, Thermo Fisher Scientific) was used to associate the observed MS/MS spectra with the proteins. The discovered proteins were examined and displayed using Perseus (version 1.6.13) (Tyanova et al., 2016; Tyanova & Cox, 2018). Using a false discovery rate (FDR) based on the Benjamini‐Hochberg method and one‐way analysis of variance (ANOVA) with a significance level of 0.05, significantly altered protein expression among SH‐exposed WT mice, SH‐exposed 5XFAD mice, and EE‐exposed 5XFAD mice were analyzed. Proteome Discoverer (Version 2.2, Thermo Fisher Scientific), DAVID bioinformatics resources (Huang da et al., 2009; Sherman et al., 2022), and the STRING database (https://string‐db.org/) were used to perform Gene Ontology (GO) annotation or protein–protein interaction (PPI) networks. We used KEGG Mapper (https://www.genome.jp/kegg/mapper/) to reveal the molecular interaction of proteome. The GraphPad Prism 9.0 program also provides statistical analysis for protein expression levels. One‐way analysis of one‐way ANOVA by Tukey's test was performed to compare three mouse groups. One‐way ANOVA and Tukey's post hoc tests were considered statistically significant at a *p*‐value of <0.05.

### 2.10 Neurotransmitter‐related analyses

After the EE exposure, mice were anesthetized with an intraperitoneal injection of Avertin at a dose of 250 μg/kg, and then the frontal cortex and hippocampus were dissected. After weighing the dissected tissue, the tissue was homogenized by adding acetonitrile containing 1% formic acid. The homogenized mixture was centrifuged at 12,000 rpm for 10 min, and the supernatant was collected and dried with nitrogen at 45°C. For injection into the LC–MS/MS system, the dried supernatant was prepared by dissolving it in 100 μL of 50% methanol containing 0.1% formic acid. The neurotransmitters and metabolites in the sampled tissues were analyzed using LC–MS/MS at NeuroVIS (Chungcheongnam‐do, Republic of Korea). An ACQUITY UPLC HSS T3 column (2.1 × 100 mm, 1.8 μm, Waters, Milford, MA, USA) at 50°C was used to separate neurotransmitters and metabolites in the LC–MS/MS system composed of ExionLC™ Series UHPLC, AB SCIEX Triple Quadrupole 6500+, and ESI (SCIEX, Framingham, MA, USA). The mobile phase consisted of (A) water containing 0.1% formic acid and 5 mM ammonium formate and (B) a ratio of 1:1 mixture of methanol and acetonitrile containing 5 mM ammonium formate. The flow rate was 0.3 mL/min, and a 10 μL per sample was injected in LS‐MS/MS. Multiple reaction monitoring (MRM) scans were performed positively or negatively for each neurotransmitter and metabolite (Table S1). The experiment was conducted at an ion transfer temperature of 500°C and a positive/negative ion spray voltage of 5000 V/−5000 V.

### 2.11 Statistical analysis

All the analyses were performed in a blinded manner for each group. Statistical analyses were performed using GraphPad Prism 9.0 (GraphPad Software Inc., La Jolla, CA, USA). The data are represented as the mean ± standard error of the mean. An independent *t*‐test was used to evaluate the significance of the differences between the two groups. Analysis ANOVA with Fisher's least significant difference test was used to determine the significance of differences among the three groups. Statistical significance was set at *p* < 0.05, which exhibited statistically significant.

## 3 RESULTS

### 3.1 EE alleviates the cognitive decline in 5XFAD mice

Previous studies have reported that EE ameliorates cognitive decline in animal models of AD, such as Samp8 and 5XFAD mice (Nakano et al., 2020; Yuan et al., 2012). To confirm the alleviatory effect of EE on cognitive deficits we conducted a behavioral test in 5XFAD mice. Spatial memory was evaluated by assessing spontaneous alternations within the Y‐maze. The SH‐exposed 5XFAD mice showed significantly lower spontaneous alterations than SH‐exposed WT mice. In contrast, 5XFAD mice exposed to EE intervention exhibited significantly increased spontaneous alterations compared to 5XFAD mice exposed to SH. (Figure 1b). The total number of arm entries was not significantly different between the groups (Figure 1c). These results indicated that EE significantly ameliorate cognitive dysfunction in animal models of AD.

### 3.2 EE inhibits the AD‐associated pathologies in Aβ‐overexpressing transgenic mice

**FIGURE 2:**
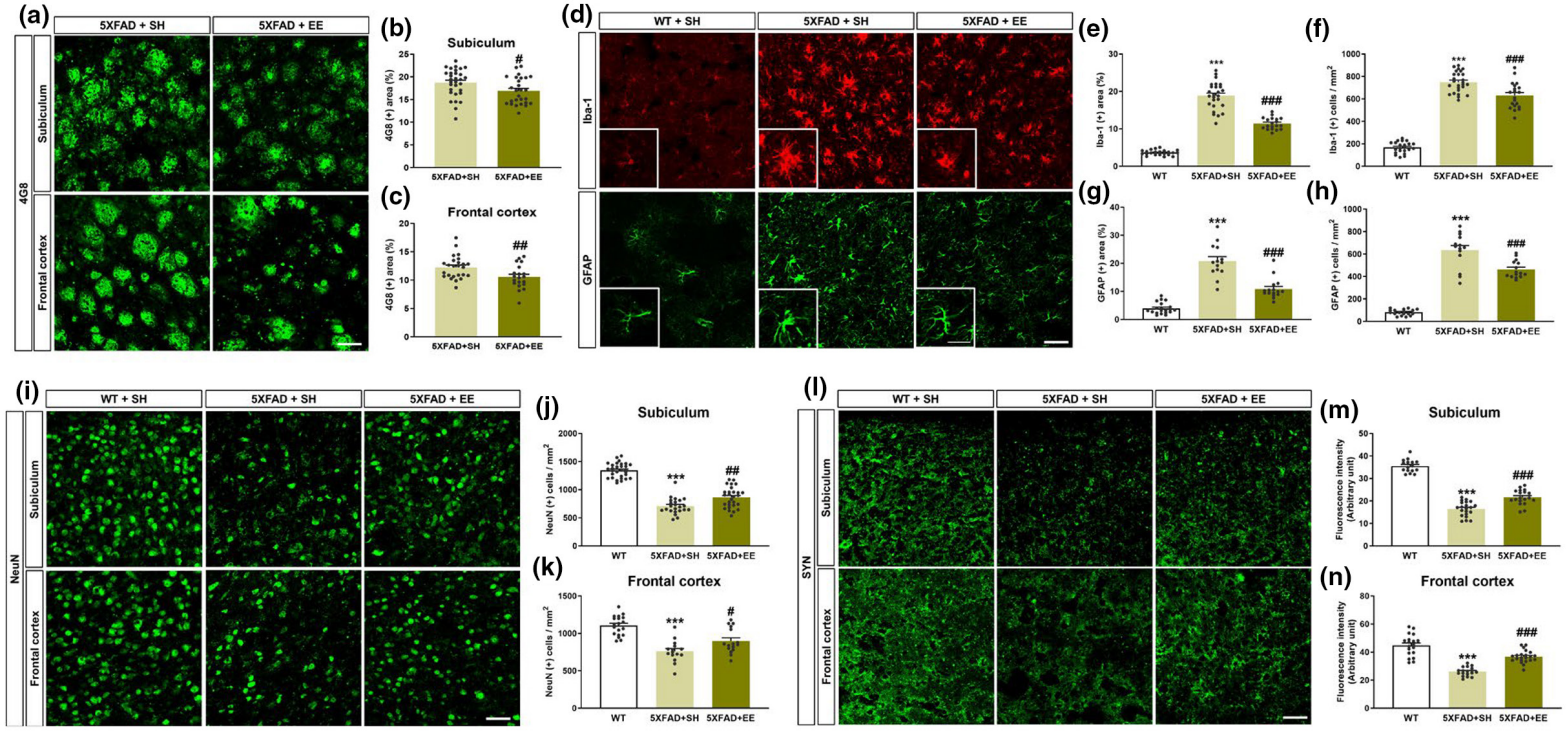
The histopathologic analysis of EE intervention in the subiculum and frontal cortex of 5XFAD mice. (a) Representative images of 4G8‐stained Aβ in the subiculum and frontal cortex of SH‐ or EE‐condition 5XFAD mice. (b) The percentage of 4G8‐positive areas in the subiculum was significantly reduced in EE‐exposed 5XFAD mice compared to SH‐exposed 5XFAD mice. (c) The percent of 4G8 (+) areas was significantly decreased in the frontal cortex of EE‐exposed 5XFAD mice compared to 5XFAD mice in SH. (d) Representative images of anti‐ionized calcium‐binding adapter molecule 1 (Iba‐1)‐ or anti‐glia fibrillary acidic protein (GFAP)‐stained section in the frontal cortex of WT and 5XFAD mice in SH or EE conditions. (e and g) The bar graphs show the quantification of the area fraction of the Iba‐1‐ and GFAP‐positive areas, respectively. (f and h) The bar graphs exhibit the number of Iba‐1 and GFAP‐positive cells in the frontal cortex, respectively. (i) Representative images of immunostained sections with the neuronal nuclei (NeuN) antibody in the subiculum and frontal cortex of WT and 5XFAD mice after SH or EE exposure. (j and k) The quantification of the number of NeuN‐positive cells in the subiculum and frontal cortex. (l) Representative images of immunostained sections with the synaptophysin (SYN) antibodies in the subiculum and frontal cortex of WT and 5XFAD mice after SH or EE exposure. (m and n) The bar graphs show the quantification of the fluorescence intensity of SYN‐positive signals in the subiculum and frontal cortex. Scale bar = 50 μm (subiculum and frontal cortex) and 25 μm (Enlarged image). Values are expressed as the mean ± S.E.M (*n* = 5 in SH‐exposed WT mice, SH‐exposed 5XFAD mice, and EE‐exposed 5XFAD mice, respectively). Statistical analysis between two or three groups was evaluated by independent *t*‐test or one‐way ANOVA, followed by Fisher's LSD test, respectively. ^#^ *p* < 0.05, ^##^ *p* < 0.01, and ^###^ *p* < 0.001 indicates significant differences compared with the SH‐exposed 5XFAD mice. ***p < 0.001 displays significant differences compared with the SH‐exposed WT mice.

We investigated changes in Aβ accumulation, neuroinflammation, neuronal cell death, and synaptic loss via immunofluorescent staining in the subiculum, frontal cortex, or both of 5XFAD mice to elucidate the histopathological mechanisms underlying the inhibitory effect of EE intervention on cognitive decline in 5XFAD mice (Figure 1a). The antibodies against 4G8, Iba‐1, GFAP, NeuN, and SYN were used to stain Aβ, microglia, astrocytes, neuronal cells, and pre‐synaptic vesicles, respectively. Immunoreactivity of the 4G8 antibody revealed that EE significantly decreased Aβ plaques in the subiculum and frontal cortex of 5XFAD mice compared to SH‐exposed 5XFAD mice (Figure 2a–c). Next, we performed immunofluorescence staining to detect neuroinflammation in the frontal cortex of 5XFAD mice using Iba‐1 and GFAP antibodies (Figure 2d). The percentage of the area fraction of Iba‐1 and GFAP in the frontal cortex of SH‐exposed 5XFAD mice was significantly higher than that in SH‐exposed WT mice (Figure 2e,g). Similarly, the number of Iba‐1‐ and GFAP‐positive cells in the frontal cortex was significantly increased in the 5XFAD mice than in the WT mice (Figure 2f,h; Figure S4). The number of microglia and astrocytes was significantly decreased in the EE‐exposed 5XFAD mice than in the SH‐exposed 5XFAD mice (Figure 2f,h). Next, the subiculum and frontal cortex of 5XFAD mice were stained with anti‐NeuN and anti‐SYN antibodies to examine the loss of neurons and synapses (Figure 2i,l). The number of NeuN (+) cells and the fluorescence intensity of SYN were significantly decreased in the subiculum and frontal cortex of SH‐exposed 5XFAD mice compared to WT mice (Figure 2j,k,m,n). However, the number of NeuN (+) cells and the fluorescence intensity of SYN were significantly increased in EE‐exposed 5XFAD mice compared to those in SH‐exposed 5XFAD mice (Figure 2j,k,m,n). Taken together, these results demonstrated that the memory‐enhancing effect of EE intervention in AD can be mediated by ameliorating AD‐related histopathologies in the brain with AD.

### 3.3 EE changes the synapse‐related proteome in the brain of 5XFAD mice

**FIGURE 3:**
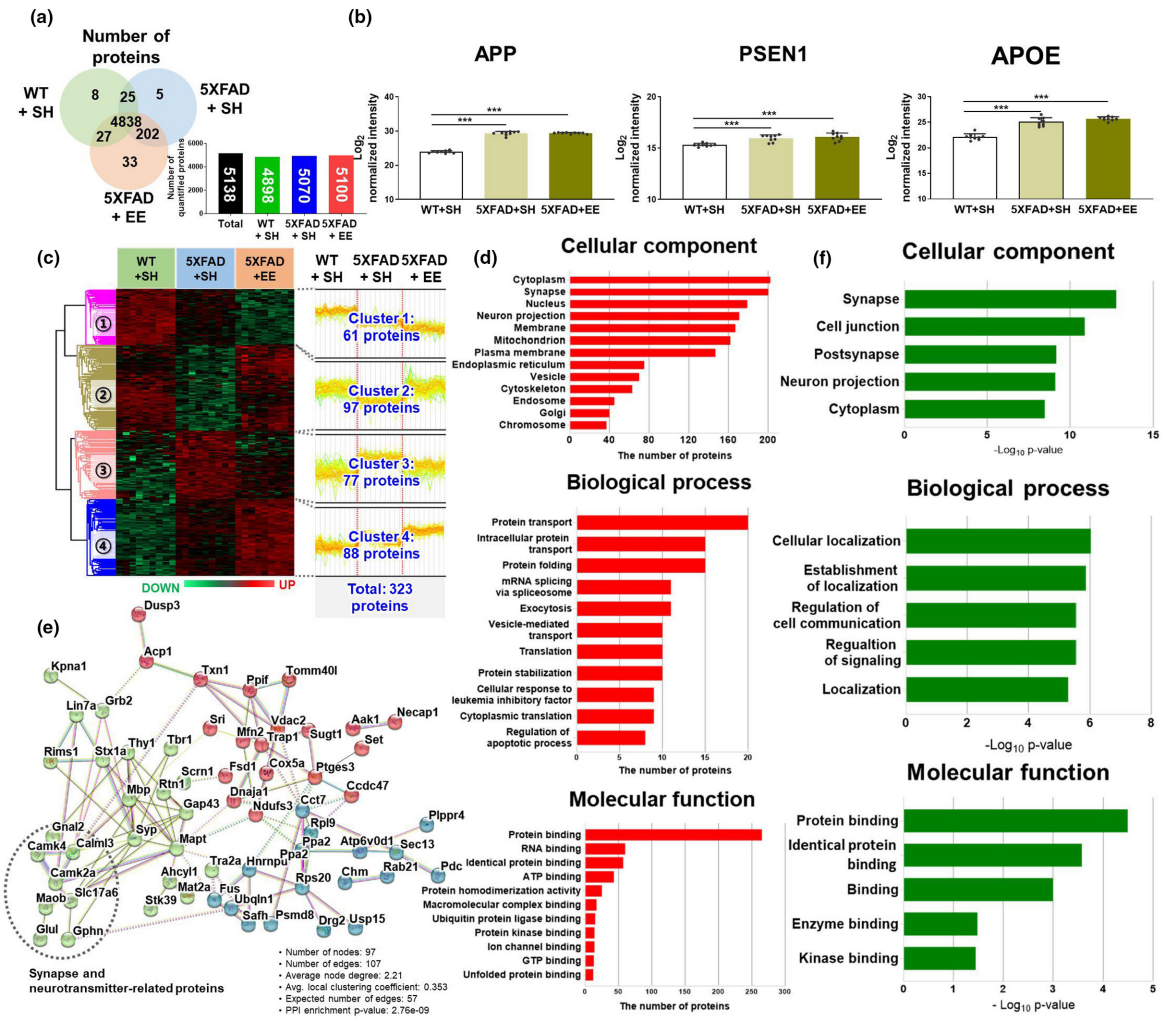
The proteomic analysis in the frontal cortex of WT and 5XFAD mice in SH or EE exposure. (a) Venn diagrams and bar graphs represent the protein levels among the three groups. (b) Three AD‐associated proteins (APP, PSEN1, and APOE) were assessed as positive controls in all three groups. Values are expressed as the mean ± SEM (*n* = 5 in SH‐exposed WT mice; *n* = 5 in SH‐exposed 5XFAD mice; and *n* = 5 in EE‐exposed 5XFAD mice). Statistical analysis between three groups was evaluated by one‐way ANOVA, followed by Fisher's LSD test. ***p < 0.001 indicates significant differences compared with the SH‐exposed WT mice. Comparative analysis of differentially expressed proteins (DEPs) in the frontal cortex of WT and 5XFAD mice under EE and SH conditions. (c) Hierarchical clustering of DEPs among the three groups. The level of protein expression is derived via Z‐score normalization. The DEPs were grouped into four clusters. (d) Gene Ontology (GO) analysis utilizing the Uniprot Database and DAVID bioinformatics resources for 323 proteins exhibiting changed levels of expression among the three mouse groups. The value of the *x*‐axis represents the number of proteins. (e) The network shows the protein–protein interactions (PPIs) of 97 proteins in cluster 2. Three clusters were detected by *K*‐means clustering analysis. The red, green, and blue bubbles indicated clusters 1, 2, and 3 individually. (f) GO analysis utilizing the STRIGN database for 97 proteins in cluster 2. The value of the x‐axis represents −Log_10_ (p‐value).

To investigate which specific molecular factors contribute to the mitigation of AD‐related pathology mediated by EE intervention in AD, we conducted a proteomic analysis in the WT and 5XFAD mice after SH or EE exposure (Figure 1a). Proteins were extracted from the frontal cortex of SH‐exposed WT, SH‐exposed 5XFAD, and EE‐exposed 5XFAD mice and analyzed using nano‐LC‐ESI‐MS/MS. A total of 5138 proteins were identified: 4898 proteins in SH‐exposed WT mice, 5070 proteins in SH‐exposed 5XFAD mice, and 5100 proteins in EE‐exposed 5XFAD mice. Interestingly, 4838 proteins were identified in all three groups. The probability of a protein to be assigned with MS peaks was greater than 99%, and the probability of a peptide to be assigned with MS peaks was greater than 95% (Figure 3a; Table S2). Proteome analysis was performed, and 4838 proteins were identified reproducibly in all groups. In addition, we observed that AD‐related proteins, such as APP, PSEN1, and APOE, were significantly increased in 5XFAD mice compared to WT mice (Figure 3b). These proteins were evaluated as positive controls in a previous proteomic study in 5XFAD mice (Kim et al., 2019). These results support the accuracy of our quantitative analysis.

Next, to determine significant differences between the EE interventions, we used ANOVA and pairwise comparative analyses to investigate 4838 proteins. We identified 3069 proteins significantly differentially expressed among the three groups (ANOVA, Benjamini‐Hochberg‐based FDR <0.05). Among the 3096 proteins, pairwise comparative analysis revealed (1) 2042 differentially expressed proteins (DEPs; *p* < 0.05, fold change >1.5) between SH‐exposed WT and 5XFAD mice, and (2) 422 DEPs (*p* < 0.05, fold change >1.5) between SH‐exposed 5XFAD and EE‐exposed 5XFAD mice (Table S3). We extracted 323 overlapping DEPs using ANOVA and pairwise comparative analyses. A heat map of the 323 DEPs in the frontal cortex revealed four clusters (Figure 3c; Table S4). Clusters 1 and 2 showed lower protein expression levels based on the genotype, while Clusters 3 and 4 showed higher protein expression levels based on the genotype. In addition, in Clusters 2 and 3, the protein expression that was altered by the genotype was changed by the intervention to be similar to that of the WT group. In contrast, Clusters 1 and 4 further decreased or increased the expression of proteins that were decreased or increased by genotype through intervention, respectively. Thus, proteins belonging to Clusters 2 and 3 may serve as therapeutic targets of EE intervention. Moreover, Clusters 1 and 4 may contribute to strengthening the compensatory and recovery mechanisms observed in AD. For instance, in cluster 1, the phospholipase DDHD1 (Ddhd1) and Ras‐related protein Ral‐A (Rala), which induce mitochondrial fission (Baba et al., 2014; Kashatus et al., 2011), were further downregulated by EE intervention. In addition, in Cluster 4, the Fabp5 protein, which contributes to improved cognitive function by activating the nuclear receptor peroxisome proliferator‐activated receptor β/δ PPARβ/δ pathway (Yu et al., 2014), was further upregulated by EE intervention.

We performed GO analysis using DAVID bioinformatics resources to examine the cellular components, biological processes, and molecular functions of the 323 DEPs included in four clusters. The GO terms related to 323 DEPs revealed their primary associations with cellular components, such as cytoplasm (13.0%) and synapses (12.9%); biological processes like protein transport (4.77%) and protein folding (3.58%); and molecular functions, including protein binding (37.22%) and RNA binding (8.43%) (Figure 3d). The results of GO terms related to 323 DEPs demonstrated that the expression of proteins in cytoplasm and synapses, which are involved in protein transport and binding, is markedly altered by genotype and intervention.

To further explore the cellular processes and pathways, we used the STRING database, an analytical tool for functional protein association networks. Based on the results of GO terms, we selected Cluster 2 among the 4 clusters, which showed major associations with synapses (Figure S5). Thus, we confirmed the PPI networks of Cluster 2 using the STRING database. There were three K‐means clusters, notably the green cluster, containing proteins related to synaptic function and neurotransmitter transport, indicating strong interactions among these three clusters (PPI enrichment *p*‐value <1.0e‐16, Figure 3e). GO analysis revealed that the protein network was associated with several key cellular components (Figure 3f). Subsequently, we analyzed the Kyoto Encyclopedia of Genes and Genomes (KEGG) pathway to investigate the proteins further directly linked to synapse and neurotransmitter function. KEGG is a reference knowledge base that links the genomes to biological systems. We found that eight proteins–Slc17a6, Glul, Gnai2, Gphn, Camk2a, Camk4, Maob, and Calml3–specifically formed strong molecular interactions related to synapse and neurotransmitter functions (Figure 3e). These results demonstrate that EE exposure may enhance cognitive function in AD through increasing molecular interactions between synapses and neurotransmitter‐related proteins (Figure S6). Our results showed that the cognitive restoration effect of EE intervention might be partially mediated by the modulation of the synapse‐ and neurotransmitter‐associated proteome in the brains of AD.

### 3.4 EE restores the altered level of neurotransmitters and metabolites in the brain of 5XFAD mice

**FIGURE 4:**
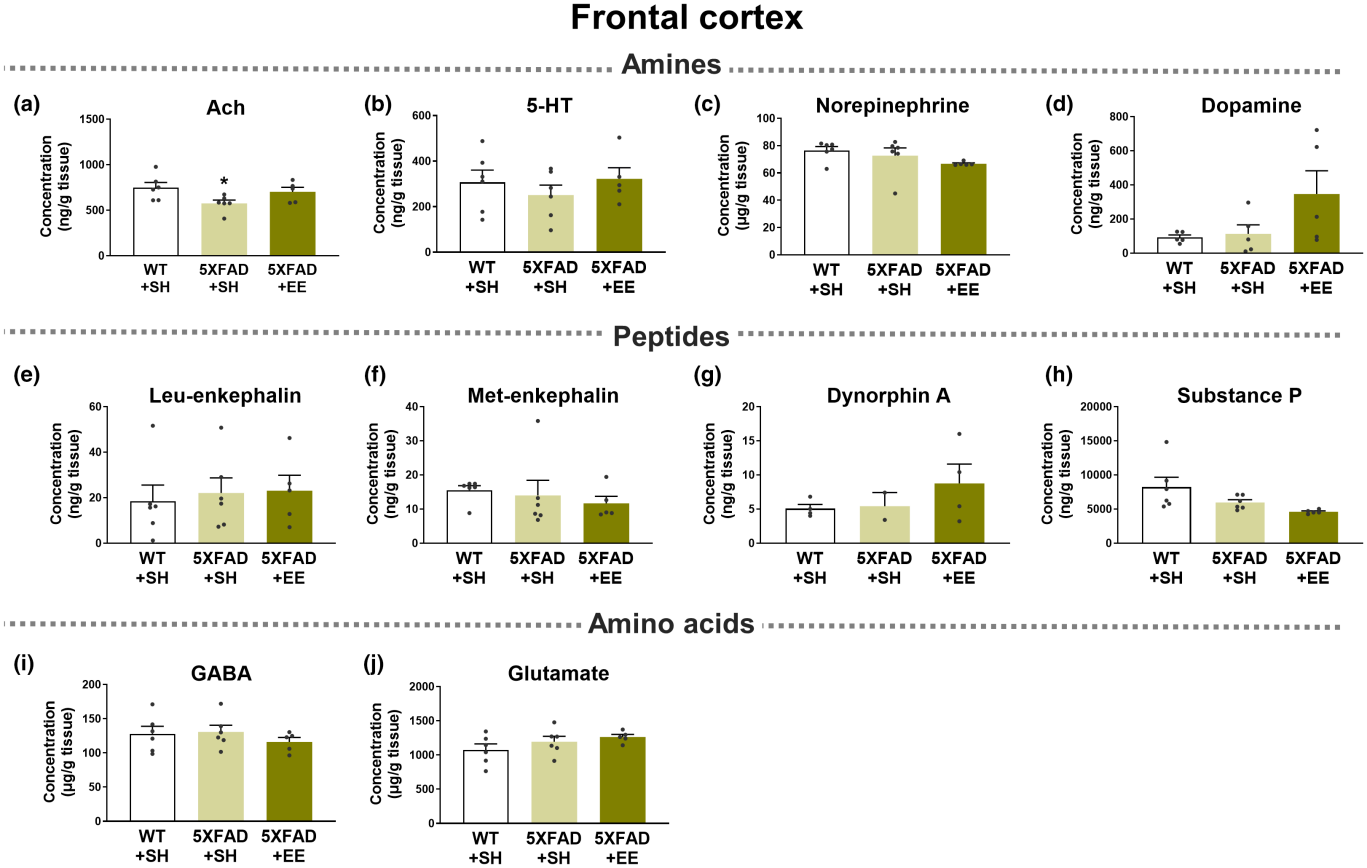
Neurotransmitter‐related analyses in the frontal cortex of WT and 5XFAD mice in SH or EE. (a) Acetylcholine (Ach), (b) 5‐Hydroxytryptamine (5‐HT, also known as serotonin), (c) Norepinephrine, (d) Dopamine, (e) Leu‐enkephalin, (f) Met‐enkephalin, (g) Dynorphin A, (h) Substance P, (i) γ‐aminobutyric acid (GABA), and (j) Glutamate were estimated in the frontal cortex of WT and 5XFAD under EE and SH conditions using LC–MS/MS. Values are expressed as the mean ± SEM (*n* = 6 in SH‐exposed WT mice; *n* = 6 in SH‐exposed 5XFAD mice; and *n* = 5 in EE‐exposed 5XFAD mice). Statistical analysis between three groups was evaluated by one‐way ANOVA, followed by Fisher's LSD test. *p < 0.05 displays significant differences compared with the SH‐exposed WT mice.

**FIGURE 5:**
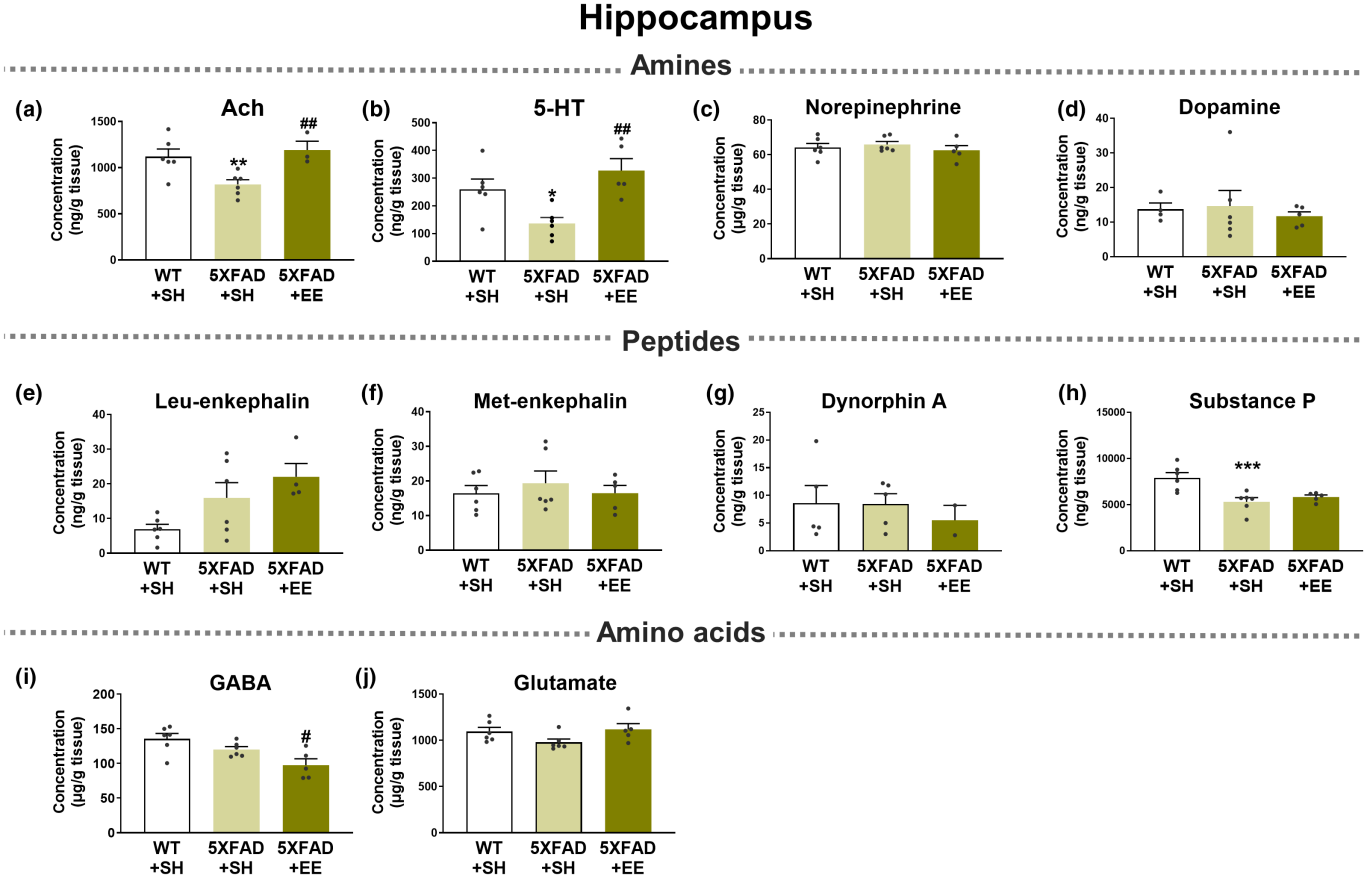
Neurotransmitter‐related analyses in the hippocampus of WT and 5XFAD mice under SH or EE. (a) Acetylcholine (Ach), (b) 5‐Hydroxytryptamine (5‐HT, also known as serotonin), (c) Norepinephrine, (d) Dopamine, (e) Leu‐enkephalin, (f) Met‐enkephalin, (g) Dynorphin A, (h) Substance P, (i) γ‐aminobutyric acid (GABA), and (j) Glutamate were estimated in the hippocampus of WT and 5XFAD under EE and SH conditions using LC–MS/MS. Values are expressed as the mean ± SEM (*n* = 6 in SH‐exposed WT mice; *n* = 6 in SH‐exposed 5XFAD mice; and *n* = 5 in EE‐exposed 5XFAD mice). Statistical analysis between two or three groups was evaluated by one‐way ANOVA, followed by Fisher's LSD test. *p < 0.05, **p < 0.01, and ***p < 0.001 display significant differences compared with the SH‐exposed WT mice. ^#^ *p* < 0.05 and ^##^ *p* < 0.01 indicate significant differences compared with the SH‐exposed 5XFAD mice.

The pathogenesis of AD is closely related to an imbalance in neurotransmitters (NTs), such as acetylcholine (Ach), dopamine, gamma‐aminobutyric acid (GABA), and serotonin (5‐hydroxytryptamine; 5‐HT) [21, 22]. Indeed, the levels of Ach and GABA have been reported to be decreased in the cerebrospinal fluid (CSF) and brain of AD patients [23–25], as well as reduced 5‐HT levels in the postmortem brains of patients with AD. Notably, changes in some synapse‐ and neurotransmitter‐related proteins in AD brains were restored to normal levels after EE intervention (Figure 3e; Figure S6). Thus, we conducted a neurotransmitter‐related analysis in WT and 5XFAD mice after SH or EE exposure to investigate the changes in neurotransmitters and metabolites levels according to EE intervention in AD brains (Figures 4 and 5; Table 1).

**TABLE 1:** Effects of EE on neurotransmitters in WT and 5XFAD mice under SH or EE conditions.

Neurotransmitter			WT+SH	5XFAD+SH	5XFAD+EE
*Frontal cortex*					
Amine	Acetylcholine	Ach^b^	747.93 ± 56.35	574.73 ± 36.89*	701.36 ± 50.07
	Serotonin	5‐HT^b^	307.43 ± 53.13	251.23 ± 43.48	322.04 ± 49.44
	Catecholamines	Norepinephrine^b^	76.45 ± 27.26	72.60 ± 5.66	66.73 ± 0.61
		Dopamine^b^	92.00 ± 14.04	113.32 ± 52.56	326.24 ± 135.96
Peptides	Enkephalin	Leu‐enkephalin^b^	18.43 ± 7.08	22.03 ± 6.62	23.08 ± 6.74
		Met‐enkephalin^b^	15.50 ± 1.36	13.97 ± 4.47	11.64 ± 2.08
	Dynorphin	Dynorphin A^b^	5.05 ± 0.62	5.40 ± 2.00	8.75 ± 2.85
	Substance P	Substance P^b^	8212.33 ± 1445.59	5960.37 ± 402.47	4592.88 ± 132.87
Amino acids	GABA	GABA^a^	127.94 ± 10.92	130.70 ± 9.71	116.13 ± 6.40
	Glutamate	Glutamate^a^	1074.95 ± 87.05	1194.89 ± 77.89	1262.67 ± 37.42
*Hippocampus*					
Amine	Acetylcholine	Ach^b^	1119.47 ± 82.44	818.27 ± 50.66**	998.84 ± 128.68^##^
	Serotonin	5‐HT^b^	259.73 ± 37.13	136.53 ± 21.39*	327.88 ± 42.76^##^
	Catecholamines	Norepinephrine^b^	64.15 ± 2.33	65.88 ± 1.75	62.59 ± 2.67
		Dopamine^b^	13.70 ± 1.81	14.60 ± 4.52	11.68 ± 1.29
Peptides	Enkephalin	Leu‐enkephalin^b^	6.87 ± 1.46	15.93 ± 4.42	22.00 ± 3.84
		Met‐enkephalin^b^	16.47 ± 2.21	19.37 ± 3.53	16.48 ± 2.24
	Dynorphin	Dynorphin A^b^	8.60 ± 3.18	8.44 ± 1.87	5.50 ± 2.70
	Substance P	Substance P^b^	7911.50 ± 561.08	5307.33 ± 444.26***	5823.28 ± 218.72
Amino acids	GABA	GABA^a^	135.38 ± 7.92	120.05 ± 4.22	97.63 ± 9.12^#^
	Glutamate	Glutamate^a^	1094.52 ± 45.91	978.82 ± 34.96	1118.96 ± 61.95

Abbreviations: 5‐HT, 5‐Hydroxytryptamine; Ach, acetylcholine; EE, environmental enrichment; GABA, γ‐aminobutyric acid; SH, standard housing; WT, wild type.

^a^ μg/g tissue.

^b^ ng/g tissue.

* *p* < 0.05.

** *p* < 0.01.

*** *p* < 0.001 indicates significant differences compared with the SH‐exposed WT mice.

^#^ *p* < 0.05.

^##^ *p* < 0.01.

^###^ *p* < 0.001 indicates significant differences compared with the SH‐exposed 5XFAD mice.

The concentrations of neurotransmitters, precursors, and metabolites in the frontal cortex (Figure 4; Figure S7; Table 1; Table S5) and hippocampus (Figure 5; Figure S8; Table 1; Table S5) were analyzed using LC–MS/MS. The concentration of Ach in the frontal cortex and hippocampus was significantly decreased in SH‐exposed 5XFAD mice compared to SH‐exposed WT mice (Figures 4a and 5a). Interestingly, the concentration of Ach showed a tendency to increase in the frontal cortex of 5XFAD mice exposed to EE conditions compared to SH‐exposed 5XFAD brains (Figure 4a). Moreover, the concentration of Ach significantly increased in the hippocampus of 5XFAD mice exposed to EE compared to that in the hippocampus of SH‐exposed 5XFAD mice (Figure 5a). Interestingly, the levels of choline, a precursor of Ach, tended to decrease in the frontal cortex and hippocampus of 5XFAD mice exposed to EE compared with SH‐exposed 5XFAD mice (Figures S7a and S8a).

The concentration of 5‐HT showed a tendency to decrease in the frontal cortex of SH‐exposed 5XFAD mice compared to SH‐exposed WT mice (Figure 4b). Notably, there was a significant decrease in 5‐HT levels within the hippocampus of SH‐exposed 5XFAD mice compared to WT mice. However, EE intervention in 5XFAD mice induced a significant increase in the concentration of 5‐HT in the hippocampus compared with that in SH‐exposed 5XFAD mice (Figure 5b). There was no significant difference in the concentration of 5‐hydroxyindoleacetic acid (5‐HIAA), a metabolite of 5‐HT, between the groups; however, there was a trend for the 5‐HIAA level to decrease in EE‐exposed 5XFAD mice compared to other groups (Figures S7b and S8b). In addition, the concentrations of catecholamine neurotransmitters, such as norepinephrine and dopamine, and their metabolites did not significantly change to normal levels despite EE intervention (Figures 4c,d and 5c,d; Figures S7c–f and S8c–f).

In the frontal cortex, polyamines, such as putrescine, spermidine, and spermine, were significantly increased in SH‐exposed 5XFAD mice compared to SH‐exposed WT mice (Figure S7g–i). Surprisingly, the frontal cortex of EE‐exposed 5XFAD mice exhibited significantly decreased levels of polyamines compared to SH‐exposed 5XFAD mice (Figure S7g–i; Table S5). Furthermore, polyamines were significantly increased in the hippocampus of Aβ‐overexpressing transgenic mice compared to WT mice and decreased after EE exposure (Figure S8g–i; Table S5). After the EE intervention, unfortunately, there were no significant differences in neurotransmitter concentrations, including γ‐aminobutyric acid, glutamate, aspartate, enkephalin, dynorphin, and substance P (Figures 4e–j and 5e–j; Figures S7j–k and S8j–k). Taken together, brain stimulation with EE normalized the altered concentrations of Ach, 5‐HT, and polyamines in the brains of patients with AD.

## 4 DISCUSSION

**FIGURE 6:**
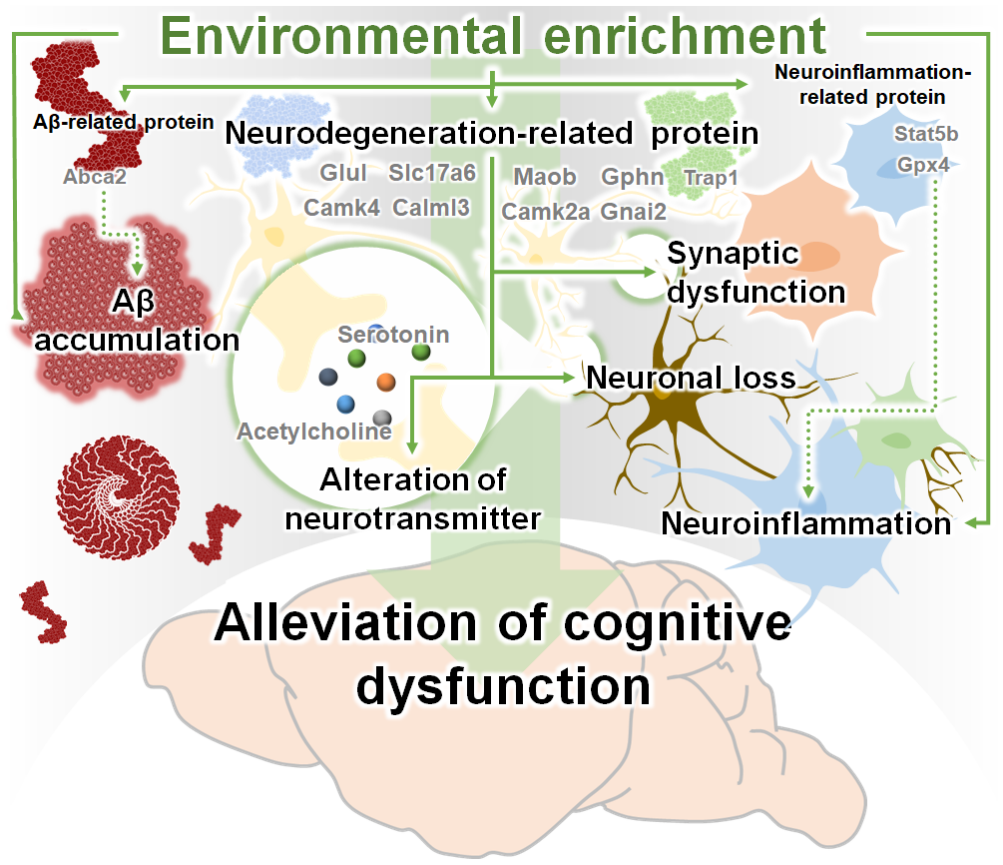
Schematic diagram of the therapeutic mechanisms of EE on cognitive impairment in AD. The therapeutic mechanisms of EE are: 1) attenuation of AD‐related pathologies, including Aβ accumulation, neuroinflammation, and neurodegeneration, 2) upregulation of the synaptic and neurotransmitter‐related proteome, and 3) modulation of neurotransmitter concentrations in the AD brain. Consequently, EE intervention alleviates cognitive impairment in AD.

Several studies have reported that EE intervention alleviates memory loss and reduces Aβ deposition and hyperphosphorylated tau in AD (Herring et al., 2009; Lahiani‐Cohen et al., 2011; Lazarov et al., 2005; Yang et al., 2021). However, there have been no studies on the therapeutic mechanisms of EE intervention in alleviating AD symptoms. We performed three mechanistic analyses to investigate the therapeutic mechanism: neurohistologic, proteomic, and neurotransmitter‐related analysis. First, we confirmed the mitigating effect of EE cognitive dysfunction in the Aβ‐overexpressing transgenic mice model of AD (Figure 1). Next, we examined the neurohistologic mechanism of EE exposure for the treatment of AD, and found that EE intervention alleviates Aβ deposition as well as AD‐related pathologies, such as neuroinflammation, neuronal cell death, and synaptic loss in the brain of AD animal model (Figure 2). Moreover, we performed a proteomic analysis to investigate the proteomic mechanisms of EE intervention in AD. Our proteomic analysis showed that EE intervention strengthened synaptic and neurotransmitter‐related networks and increased the expression of synaptic and neurotransmitter‐related proteins that were downregulated in AD brains (Figure 3). In particular, EE intervention changed synapse‐related proteins in AD brains to similar levels observed in WT brains. Finally, to explore the therapeutic mechanism of EE intervention in AD, we analyzed alterations in neurotransmitter and metabolites levels in the brains of 5XFAD mice using LC–MS/MS. We found that EE intervention increased the concentrations of reduced Ach and 5‐HT, and decreased the levels of elevated polyamines, such as putrescine, spermidine, and spermine, in the AD brain (Figures 4 and 5; Table 1; Table S5). In this study, we demonstrated that EE exerts a therapeutic effect on AD brains through mechanisms that (1) alleviate AD‐related neuropathology, (2) change the synapse‐ and neurotransmitter‐related proteomes, and (3) modulate the concentrations of neurotransmitters, including Ach, 5‐HT, and polyamines, which are altered by AD (Figure 6).

In addition to synapse‐ and neurotransmitter‐related proteins, some proteins in the cluster can directly contribute to ameliorating AD‐related pathologies, such as Aβ deposition, neuroinflammation, and neurodegeneration. ATP‐binding cassette sub‐family A member 2 (Abca2), present in Cluster 3, is one of the risk factors for AD, and increased expression of Abca2 promotes Aβ production (Chen et al., 2004; Mace et al., 2005). The increased expression of Abca2 in 5XFAD mice was reduced through EE intervention, and this reduction can reduce the production of Aβ and thereby inhibit the deposition of Aβ. In addition, the signal transducer and activator of transcription 5B (Stat5b), belonging to Cluster 3, causes neuroinflammation through the Stat5b‐NF‐κB pathway (Pu et al., 2022). Phospholipid hydroperoxide glutathione peroxidase 4 (Gpx4) in Cluster 4 attenuates neuroinflammation by suppressing tumor necrosis factor (TNF)‐mediated activation of NF‐ĸB signaling (Wang et al., 2022). EE intervention can alleviate neuroinflammation by regulating the increased Stat5b and Gpx4 expressions in 5XFAD mice. Moreover, tumor necrosis factor receptor‐associated protein 1 (Trap1), also known as heat shock protein 75 in Cluster 2 is involved in neurodegeneration by reducing reactive oxygen species and protecting against apoptosis (Ramos Rego et al., 2021). EE intervention can contribute to alleviating neurodegeneration by increasing the expression of Trap1 protein, which was decreased in 5XFAD mice.

The effects of EE intervention on altered neurotransmitters in healthy and disease models, including post‐traumatic stress disorder, are well known (Brenes et al., 2008; Hendriksen et al., 2010; Naka et al., 2002). Some studies have reported an increase in 5‐HT, associated with emotion and circadian rhythms, in healthy rodents' frontal cortex, prefrontal cortex, and hippocampus following EE intervention. (Brenes et al., 2008, 2009; Leger et al., 2015). In addition, noradrenaline levels, which regulate the fight‐or‐flight response, were significantly increased in the hippocampus and pons/medulla oblongata of the EE intervention group compared to the SH group (Brenes et al., 2009; Naka et al., 2002). Moreover, EE intervention increases dopamine levels, which plays a vital role in reward and movement regulation in healthy brains' nucleus accumbens and striatum (Bowling et al., 1993; Segovia et al., 2010). Considering the role of EE intervention in the modulation of neurotransmitters in the healthy brain, we speculated that EE intervention might also regulate neurotransmitter levels in the AD brain. Therefore, for the first time, we conducted a neurotransmitter‐related analysis of AD brains exposed to EE intervention to investigate the therapeutic mechanisms underlying the memory‐enhancing effect of EE intervention in AD.

Interestingly, in the neurotransmitter‐related analysis of the AD brain, we revealed that EE intervention increased the concentrations of Ach and 5‐HT, which decreased in the AD brain (Figures 4 and 5, Table 1). Moreover, EE exposure reduced the abnormally elevated concentrations of polyamines in AD brains to levels similar to those in normal brains (Figures S7g–i and S8g–i; Table S5). Ach, 5‐HT, and polyamines are neurotransmitters associated with cognitive functions (Makletsova et al., 2022; Newman et al., 2012; Svob Strac et al., 2016). In summary, while EE intervention did not have a significant impact on the regulation of other neurotransmitters, such as catecholamines, glutamate, GABA, and enkephalin, the analysis of neurotransmitter‐related analysis offers insights into the therapeutic mechanism by which EE intervention alleviates cognitive dysfunction through the regulation of Ach, 5‐HT, and polyamines levels in the brain of patients with AD.

Several studies have reported that stimulating sensory inputs, such as the auditory, olfactory, and visual senses, alleviates neuropsychiatric symptoms, cognitive dysfunction, and circadian dysregulation in patients with AD (Arroyo‐Anllo et al., 2013; Guetin et al., 2009; Jimbo et al., 2009; Takahashi et al., 2020). NPIs are clinically used to alleviate symptoms in patients with AD (Gueib et al., 2020; Machado & Castro, 2022; Rivasseau Jonveaux et al., 2013). While NPIs have received much attention, one study reported that Snoezelen, an NPIs, aggravates behavioral symptoms in patients with AD (Goto et al., 2014). These results could be attributed to Snoezelen studies primarily targeting individuals with hypersensitivity to autism spectrum disorders and children. In contrast, patients with AD are mostly elderly and have movement disorders such as rigidity, slowness, abnormal gait, and problems in receiving sensory stimuli (Chui et al., 1994; Ditter & Mirra, 1987; Murphy, 2019; Pearce, 1974). Applying the Snoezelen method to patients with AD, as it has been applied to children and patients with autism spectrum disorders, may lead to negative outcomes owing to its failure to consider the patient's specific stage of the disease and symptoms. Therefore, nonclinical and neurobiological mechanistic studies are needed to provide patient‐specific NPIs based on the stage and symptoms. Considering our study, 8‐month‐old 5XFAD with Aβ accumulation, neuronal cell death, and cognitive dysfunction corresponds to the moderate AD stage. Therefore, applying EE to moderate patients with AD may effectively alleviate AD‐related symptoms and pathology. Our findings can serve as a basis for the scientific rationale and environmental conditions for applying EE interventions in patients with AD. Nevertheless, our study has several limitations that should be noted. We found that EE can regulate 323 DEPs in a global proteomic approach of an AD mouse model, however, among these we specifically focused on changes in specific synapse‐ and neurotransmitter‐related proteins. Therefore, further studies on other proteins that were changed are needed. First, future studies are needed to investigate whether altered proteins induce physiological changes. Second, our neurotransmitter‐related analyses are limited due to the utilization of homogenized brains. Amino acid neurotransmitters serve as both the energy source for the tricarboxylic acid cycle and malate–aspartate shuttles, as well as functioning as transmitters (Arnold & Finley, 2023; Borst, 2020). In addition, amine neurotransmitters act as hormonal regulators and bioregulators (Pearl & Zigmond, 2008). Due to the multifaceted functions of neurotransmitters, the utilization of homogenized brain samples may not sufficiently reflect the entire spectrum of neurotransmitter functions. Therefore, further studies are needed to exclusively investigate the role of neurotransmitters in synaptic transmission.

## 5 CONCLUSION

In this study, we revealed the therapeutic mechanisms underlying the cognitive‐enhancing effect of EE in AD. We investigated the therapeutic mechanisms of EE using histological, proteomic, and neurotransmitter‐related analyses in AD. We revealed that the memory‐enhancing effect of EE intervention in AD could be mediated by (1) alleviation of AD‐related neuropathology, (2) regulation of synapse‐ and neurotransmitter‐associated proteomes, and (3) modulation of neurotransmitters, such as Ach, 5‐HT, and polyamines, in the AD brain. Our findings offer insights into the therapeutic mechanisms underlying the memory‐enhancing effects of EE interventions and contribute to the accumulation of neurological evidence for NPIs.

## AUTHOR CONTRIBUTIONS

Y.K., S.K., Y.H.P., B‐H.K. and S.J.S. contributed equally to this study. Y.K., S.K., Y.H.P., B‐H.K. and S.J.S conducted the most assays and analyzed the data. S.H.L. and H.H.P. helped with animal housing and some experiments. G.J., J.L., and H‐G.K. helped with neurotransmitter analysis and interpreting data. D‐H.Y., H.S.K., and M.M conceived the project, designed the study, arranged the results and revised the manuscript. D‐H.Y., H.S.K., and M.M accepted full responsibility for the finished work, had access to the data and controlled the decision to publish. All authors have approved the final version of the manuscript.

## CONFLICT OF INTEREST STATEMENT

The authors declare that they have no competing interests.

## Supporting information


Data S1.

Table S2.

Table S3.

Table S4.

## Data Availability

All data generated or analysed during this study are included in this published article and its supplementary information files.
