# Supplementary material for: Investigating the impact of environmental enrichment on proteome and neurotransmitter‐related profiles in an animal model of Alzheimer's disease
Source: Aging Cell. 2024 Jul 1;23(9):e14231. doi: 10.1111/acel.14231 (PMC11488327; doi:10.1111/acel.14231)
Supplement: Supplementary file 1 — Data S1. [file ACEL-23-e14231-s004.docx]

**Supplementary information for**

**Investigating the impact of environmental enrichment on proteome and neurotransmitter profiles in an animal model of Alzheimer’s disease**

Yunkwon Nam^1^**^†^**, Sujin Kim^1, 2^**^†^**, Yong Ho Park^1^**^†^**, Byeong-Hyeon Kim^1^**^†^**, Soo Jung Shin^1,2^**^†^**, Seol Hwa Leem^1^, Hyun Ha Park^1^, Gukhwa Jung ^3^, Jeongbeen Lee^3^, Hyung-Gun Kim^3^, Doo-Han Yoo ^2, 4*^, Hak Su Kim ^5*^ and Minho Moon ^1,2*^

^1^ Department of Biochemistry, College of Medicine, Konyang University, 158, Gwanjeodong-ro, Seo-gu, Daejeon 35365, Korea

^2^ Research Institute for Dementia Science, Konyang University, 158, Gwanjeodong-ro, Seo-gu, Daejeon 35365, Korea

^3^ NeuroVis Inc., Cheonan 31035, Republic of Korea

^4^ Department of Occupational Therapy, Konyang University, 158, Gwanjeodong-ro, Seo-gu, Daejeon 35365, Korea

^5^ Veterans Medical Research Institute, Veterans Health Service Medical Center, Seoul 05368, Republic of Korea

**^†^** These authors have contributed equally to this work.

**Supplementary figure**


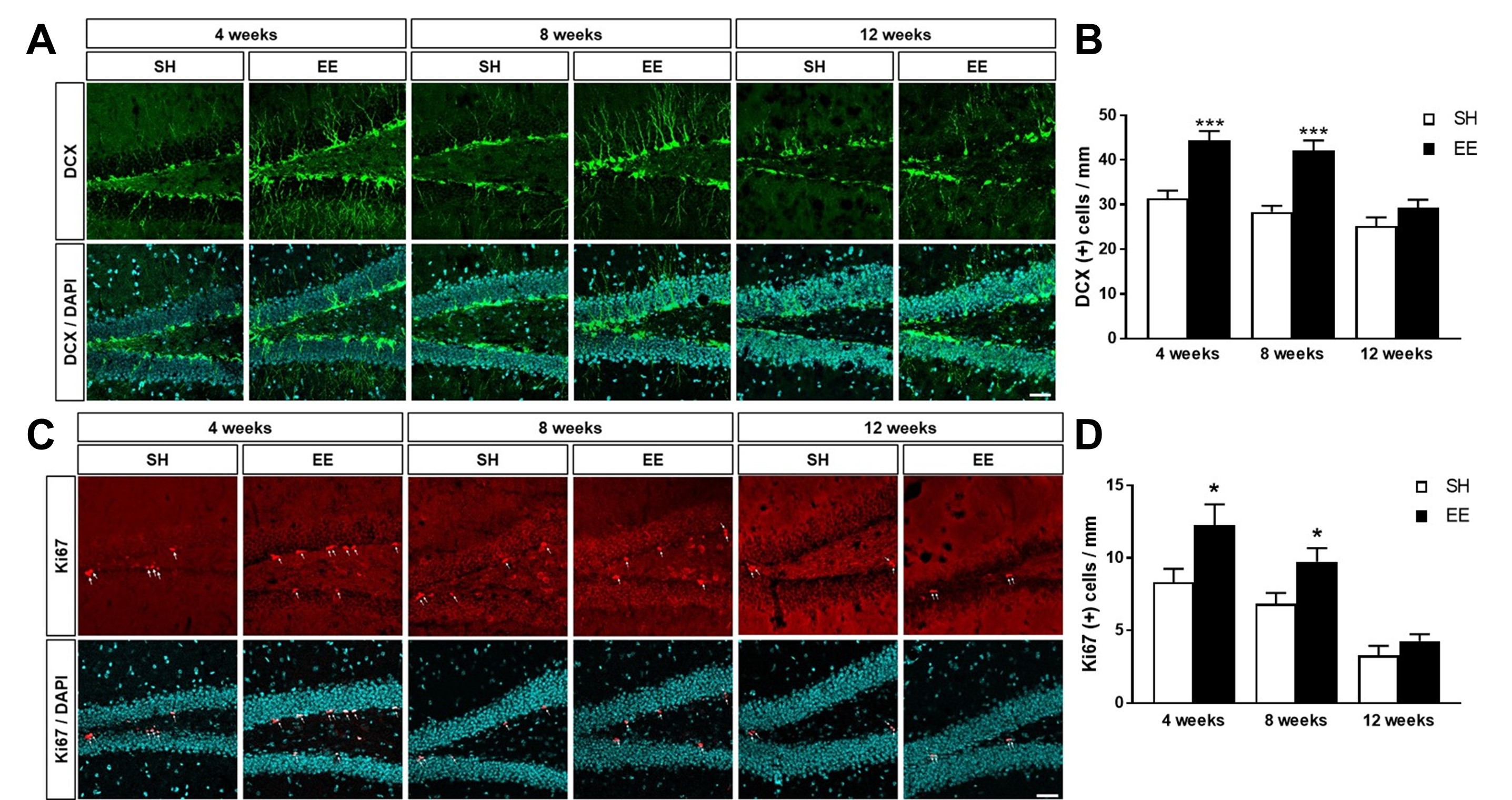


**Supplementary Figure 1.** Increased adult hippocampal neurogenesis by exposure to environmental enrichment (EE) in C57BL/6 mice. At 3-month-old mice were housed in EE or standard housing conditions in groups of 6 mice for 4, 8, and 12 weeks. (A) Representative images for doublecortin (DCX)-positive cells, marker for a neuronal precursor, used to evaluate adult hippocampal neurogenesis in the subgranular zone of the dentate gyrus. (B) The number of DCX-positive cells per length was markedly increased in the EE group compared to the SH group. (C) Representative images for Ki67-positive cells, marker for a proliferating cell, used to assess adult hippocampal neurogenesis in the subgranular zone of the dentate gyrus. (D) The number of Ki67-positive cells per length was markedly increased in the EE group compared to the SH group. Scale bar = 50 μm. Values are expressed as the mean ± S.E.M. Statistical analysis between two groups was evaluated by independent *t*-test. ^*^ *p* < 0.05, standard housing group vs. EE group.


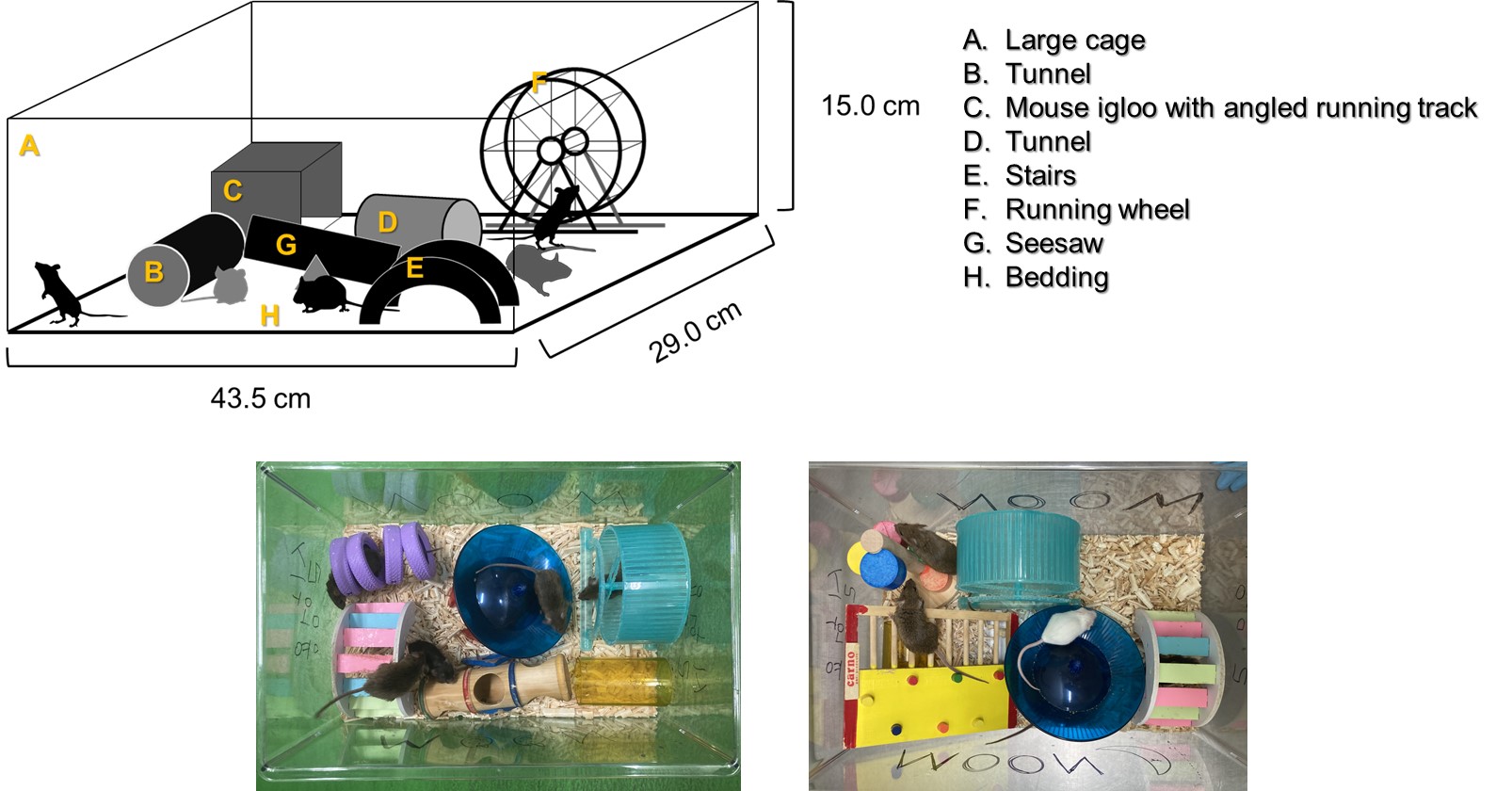


**Supplementary Figure 2.** Housing conditions of environmental enrichment (EE). Five mice were housed in EE. A running wheel, tunnel, mouse igloo, various stairs, and seesaw were placed inside a large cage.


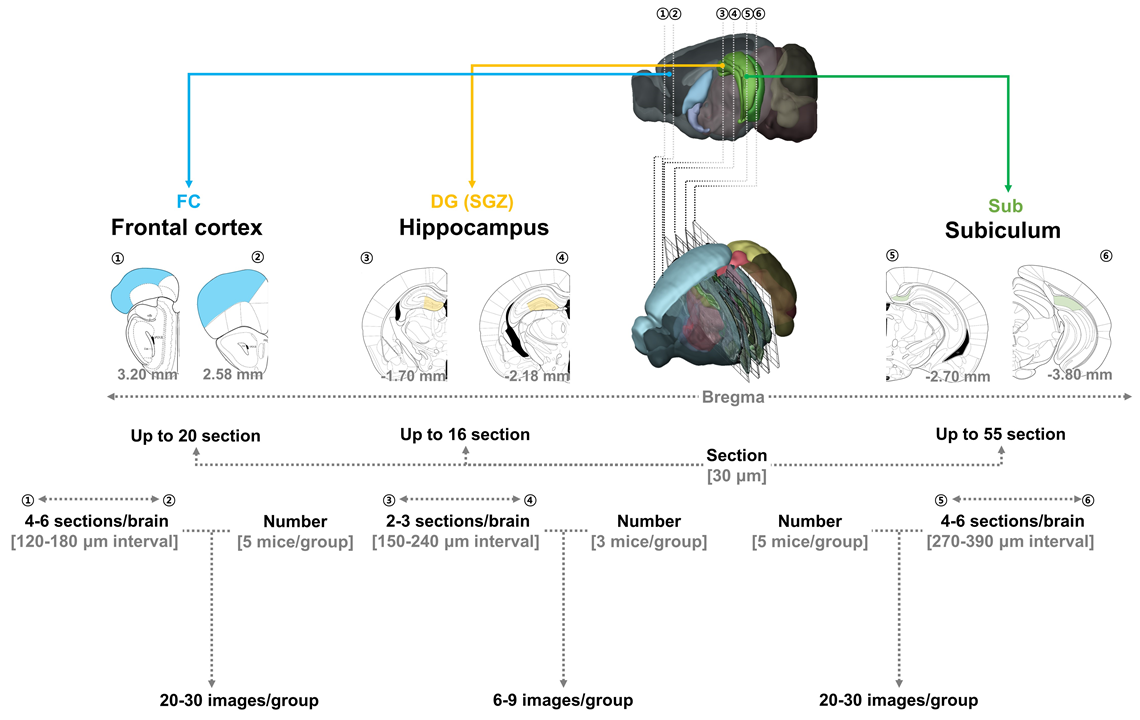


**Supplementary Figure 3.** Brain slices preparation process required for immunofluorescence staining. From 1 to 2, the frontal cortex was identified as the region between +2.58 and +3.20 mm from the bregma. From 3 to 4, the subgranular zone (SGZ) of the dentate gyrus (DG) was identified as the region between -1.70 and -2.18 mm from the bregma. From 5 to 6, the subiculum (Sub) was defined as the area extending from -2.70 to -4.36 mm from the bregma. Mouse brains were fixed, cryoprotected, and sectioned coronally at a thickness of 30 μm using a cryostat. Sixteen sections containing the SGZ of the DG and 55 sections containing the Sub were obtained per mouse. For the SGZ of DG, two to three sections were collected per brain from 3 mice at intervals of 150-240 μm, resulting in a total of 6-9 images. For the Sub and FC, four to six sections were collected per brain from 5 mice at intervals of 270-390 μm, resulting in a total of 20-30 images.
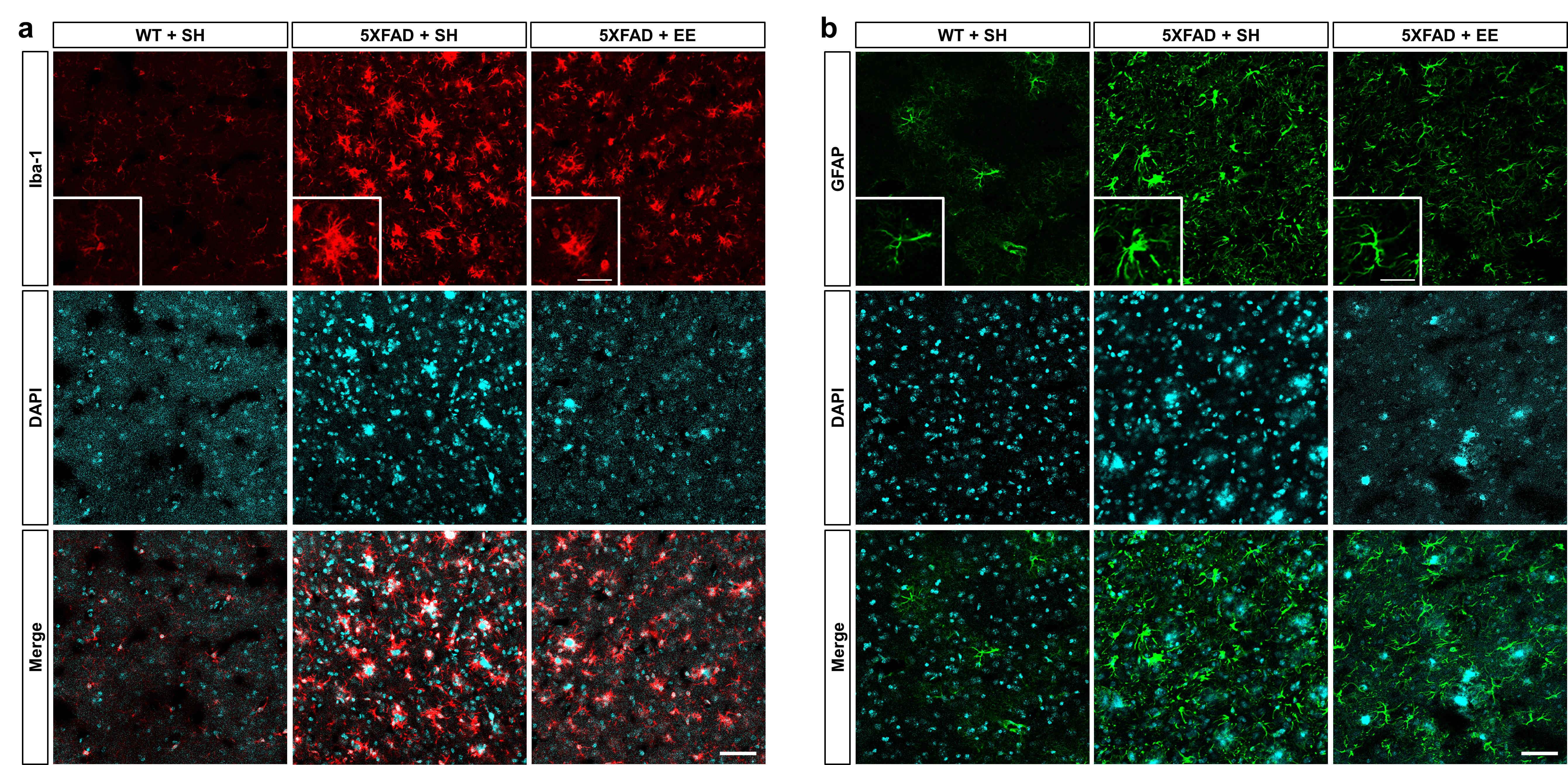


**Supplementary Figure 4**. Inhibitory effect of EE on neuroinflammation in the frontal cortex of 5XFAD mice. (a, b) Representative images of immunofluorescence staining for Iba-1 and GFAP, a marker of microglia and astroglia, in the frontal cortex. Scale bar= 50 μm (frontal cortex) and 25 μm (Enlarged image).


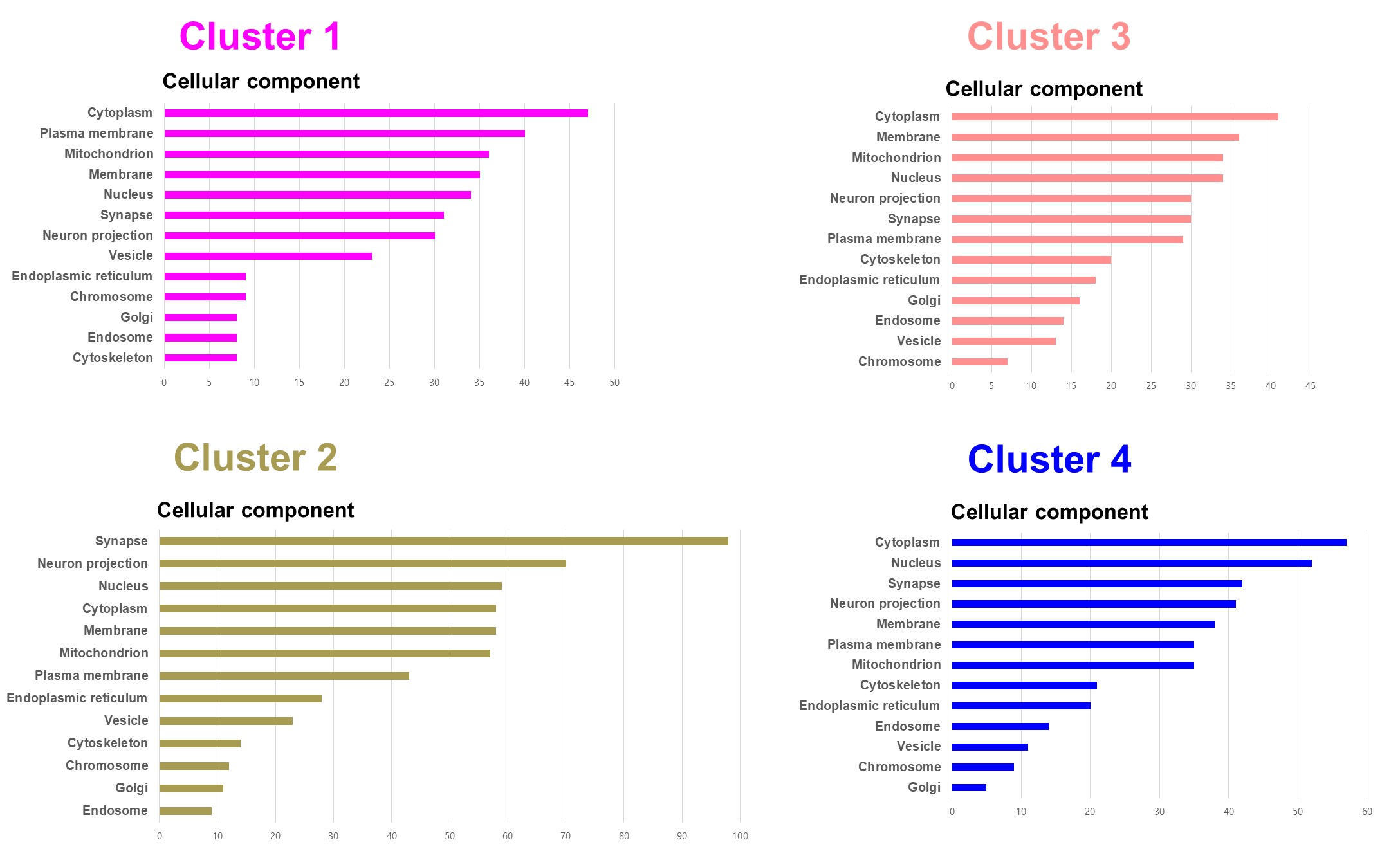


**Supplementary Figure 5.** Gene Ontology (GO) analysis of 323 DEPs belonging to four clusters. The cellular components associated with 61 DEPs belonging to cluster 1 are the cytoplasm, plasma membrane, and mitochondrion. The cellular components associated with 97 DEPs belonging to cluster 2 are the synapse, neuron projection, and nucleus. The cellular components associated with 77 DEPs belonging to cluster 3 are the cytoplasm, membrane, and mitochondrion. The cellular components associated with 88 DEPs belonging to cluster 4 are the cytoplasm, nucleus, and synapse.


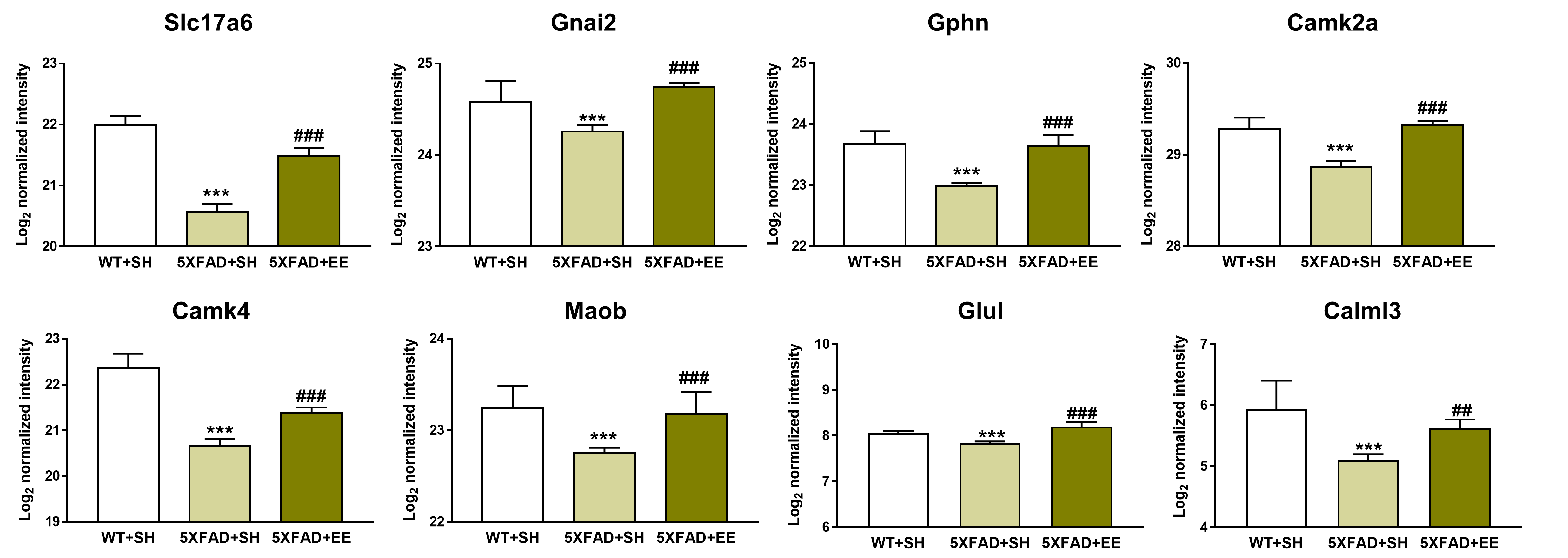


**Supplementary Figure 6.** Modulatory effects of EE on altered synapse and neurotransmitter-related proteins in AD mouse model. Bar graphs showing the abundance (y-axis) of the synapse-associated proteins from the standard housing WT mice (white bars), the standard housing 5XFAD mice (light green bars), and the EE-exposed 5XFAD mice (dark green bars). Statistical analysis between three groups was evaluated by one-way ANOVA by Fisher’s LSD test. ^***^ *p* < 0.001 indicates significant differences compared with the the standard housing WT mice. ^##^ *p* < 0.01 and ^###^ *p* < 0.001 indicates significant differences compared with the the standard housing 5XFAD mice.


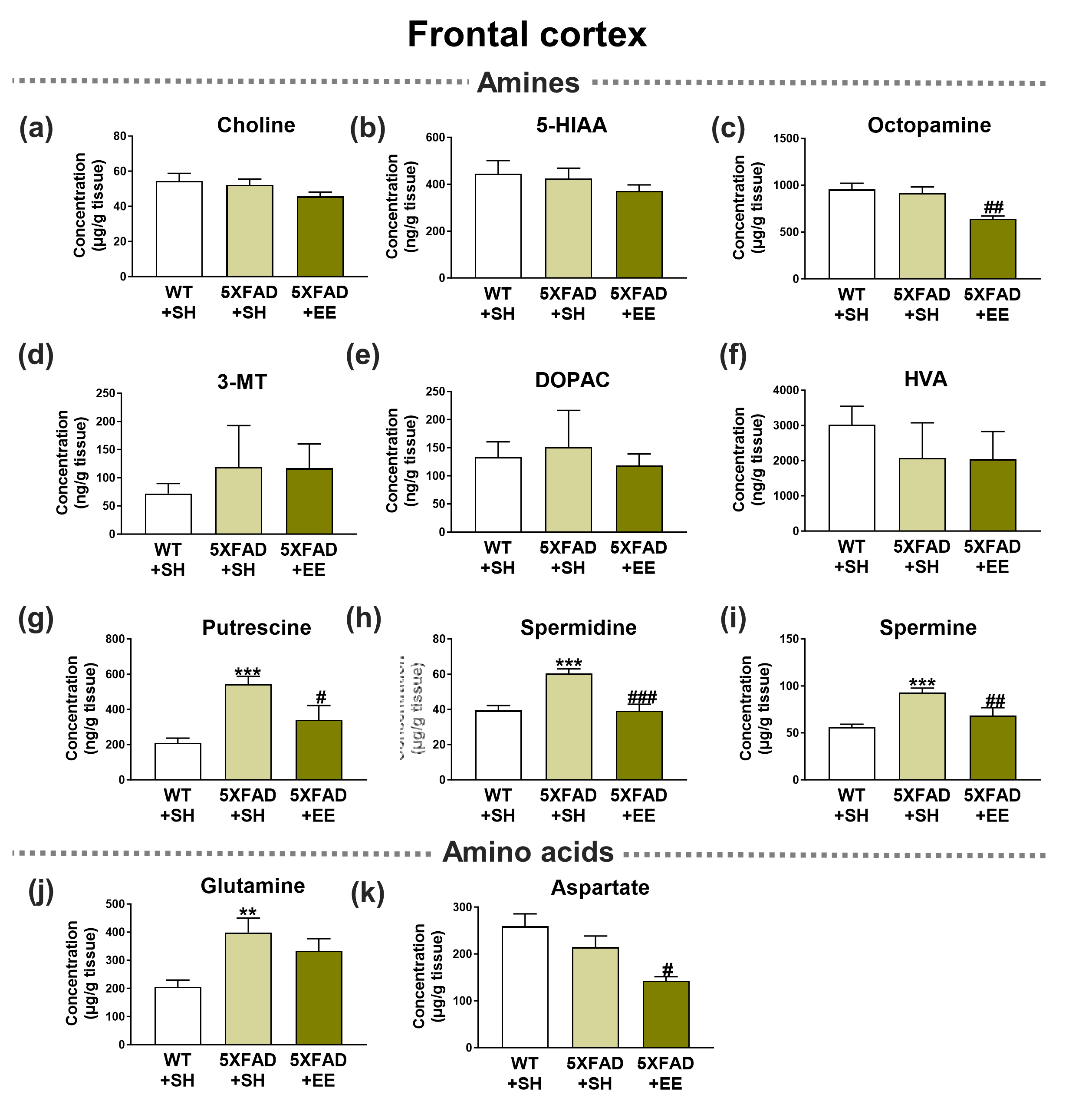


**Supplementary Figure 7.** Metabolites of neurotransmitter analysis in the frontal cortex of WT and 5XFAD mice in SH or EE. Choline (a), 5-Hydroxy indoleacetic acid (5-HIAA) (b), Octopamine (c), 3-methoxytyramine (3-MT) (d), 3,4-dihydroxyphenylacetic acid (DOPAC) (e), Homovanillic acid (HVA) (f), Putrescine (g), Spermidine (h), Spermine (i), Glutamine (j), Aspartate (k) were estimated in the frontal cortex of WT and 5XFAD under EE and SH conditions using LC-MS/MS. Values are expressed as the mean ± S.E.M (n = 6 in standard-housing WT mice; n = 6 in standard-housing 5XFAD mice; and n = 5 in EE-exposed 5XFAD mice). Statistical analysis between three groups was evaluated by one-way ANOVA, followed by Fisher’s LSD test. ^**^ *p* < 0.01, and ^***^ *p* < 0.001 displays significant differences compared with the standard-housing WT mice. ^#^ *p* < 0.05, ^##^ *p* < 0.01, and ^###^ *p* < 0.001 indicates significant differences compared with the standard-housing 5XFAD mice.


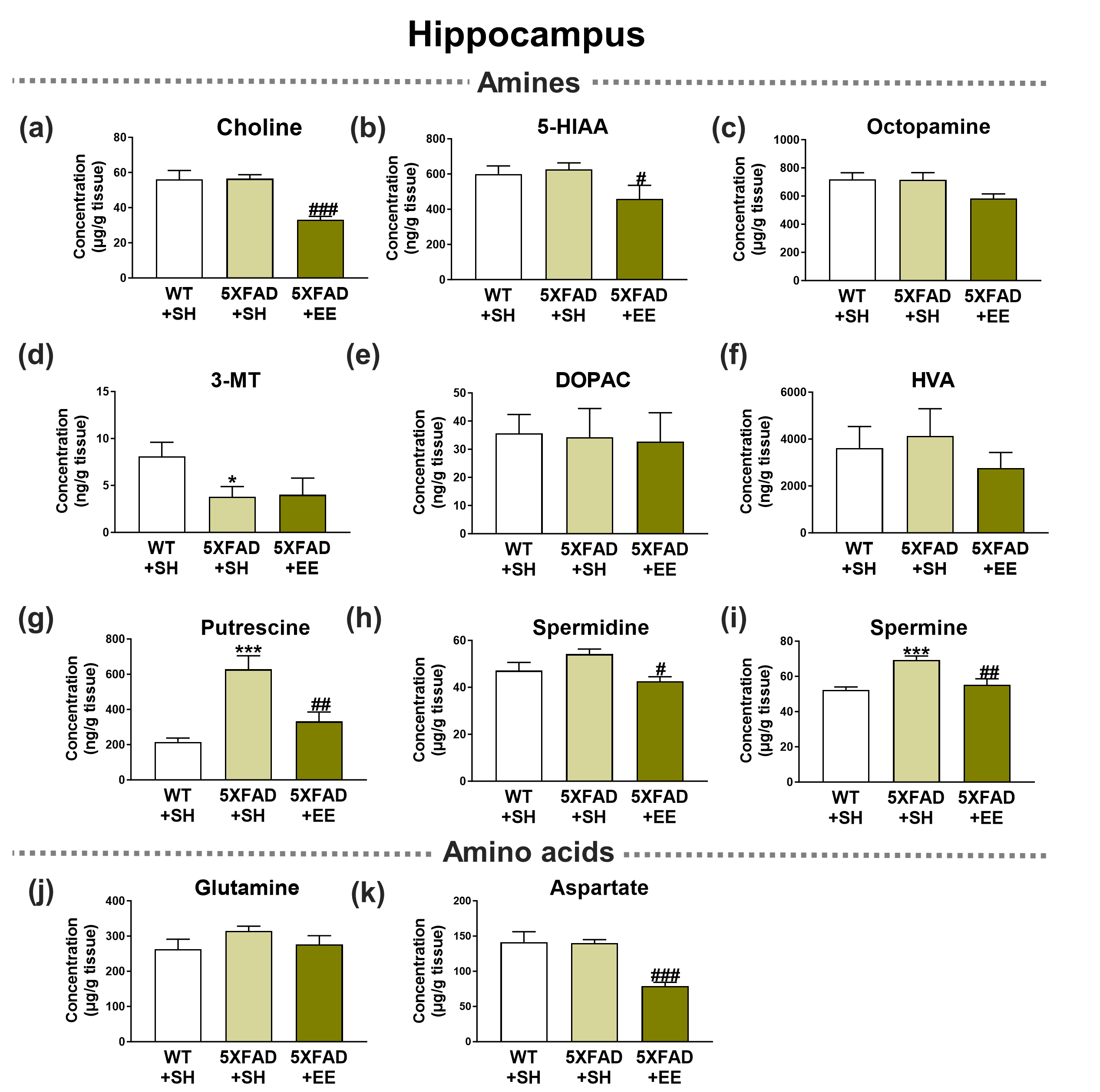


**Supplementary Figure 8.** Metabolites of neurotransmitter analysis in the hippocampus of WT and 5XFAD mice in SH or EE. Choline (a), 5-Hydroxy indoleacetic acid (5-HIAA) (b), Octopamine (c), 3-methoxytyramine (3-MT) (d), 3,4-dihydroxyphenylacetic acid (DOPAC) (e), Homovanillic acid (HVA) (f), Putrescine (g), Spermidine (h), Spermine (i), Glutamine (j), Aspartate (k) were estimated in the frontal cortex of WT and 5XFAD under EE and SH conditions using LC-MS/MS. Values are expressed as the mean ± S.E.M (n = 6 in standard-housing WT mice; n = 6 in standard-housing 5XFAD mice; and n = 5 in EE-exposed 5XFAD mice). Statistical analysis between three groups was evaluated by one-way ANOVA, followed by Fisher’s LSD test. ^*^ *p* < 0.05, and ^***^ *p* < 0.001 displays significant differences compared with the standard-housing WT mice. ^#^ *p* < 0.05, ^##^ *p* < 0.01, and ^###^ *p* < 0.001 indicates significant differences compared with the standard-housing 5XFAD mice.

**Supplementary Table 1.** Multi-reaction monitoring (MRM) condition for the analysis of neurotransmitters

| **Analyte** | **Q1 Mass (m/z)** | **Q3 Mass (m/z)** | **DP (V)** | **CE (V)** | **CXP (V)** | **Ionization polarity** |
| --- | --- | --- | --- | --- | --- | --- |
| γ-aminobutyric acid | 104 | 87 | 26 | 15 | 10 | Positive |
| Glutamate | 148 | 84.2 | 21 | 25 | 10 | Positive |
| 5-Hydroxytryptamine | 177.1 | 160.1 | 26 | 21 | 18 | Positive |
| 5-Hydroxy indoleacetic acid | 192 | 146.1 | 26 | 23 | 16 | Positive |
| Acetylcholine | 146.9 | 88.1 | 46 | 21 | 10 | Positive |
| Choline | 104.1 | 60 | 30 | 20 | 10 | Positive |
| Dopamine | 154 | 137 | 31 | 21 | 16 | Positive |
| 3-methoxytyramine | 168.074 | 151 | 1 | 13 | 16 | Positive |
| 3,4-dihydroxyphenylacetic acid | 167 | 123 | -15 | -14 | -7 | Negative |
| Homovanillic acid | 180.9 | 136.9 | -40 | -12 | -15 | Negative |
| Norepinephrine | 170 | 152.1 | 6 | 12 | 16 | Positive |
| Putrescine | 89.383 | 72 | 21 | 17 | 8 | Positive |
| Spermidine | 146.039 | 72 | 60 | 15 | 8 | Positive |
| Spermine | 203.145 | 129.1 | 40 | 15 | 8 | Positive |
| Octopamine | 154 | 136 | 6 | 11 | 6 | Positive |
| Glutamine | 147 | 84 | 1 | 18 | 10 | Positive |
| Leu-enkephalin | 556.2 | 120 | 211 | 73 | 12 | Positive |
| Met-enkephalin | 574.2 | 120 | 1 | 77 | 14 | Positive |
| Substance P | 674 | 594 | 70 | 39 | 8 | Positive |
| Aspartate | 134 | 74 | 30 | 19 | 8 | Positive |
| Dynorphin A | 491.3 | 434.8 | 116 | 29 | 12 | Positive |

**Supplementary Table 5.** Effects of EE on metabolites of neurotransmitters in WT and 5XFAD mice under SH or EE conditions.

| **Neurotransmitter** | | | **WT+SH** | **5XFAD+SH** | **5XFAD+EE** |
| --- | --- | --- | --- | --- | --- |
| ***Frontal cortex*** | | | | | |
| Amine | Acetylcholine | Choline ^a^ | 54.34 ± 4.46 | 52.09 ± 3.47 | 45.61 ± 2.58 |
|  | Serotonin | 5-HIAA ^b^ | 444.83 ± 56.33 | 424.07 ± 44.63 | 371.08 ± 26.23 |
|  | Catecholamines | Octopamine ^a^ | 954.45 ± 65.85 | 913.68 ± 67.82 | 640.83 ± 30.66 |
|  |  | 3-MT ^b^ | 72.03 ± 17.90 | 119.27 ± 73.49 | 117.04 ± 42.87 |
|  |  | DOPAC ^b^ | 133.36 ± 27.26 | 151.43 ± 64.95 | 118.04 ± 20.73 |
|  |  | HVA ^b^ | 3022.88 ± 521.11 | 2072.16 ± 1001.91 | 2041.76 ± 787.99 |
|  | Polyamines | Putrescine ^b^ | 210.44 ± 26.36 | 543.53 ± 44.32 ^***^ | 340.72 ± 81.15 ^#^ |
|  |  | Spermidine ^a^ | 39.34 ± 2.81 | 60.34 ± 2.66 ^***^ | 39.16 ± 3.71 ^###^ |
|  |  | Spermine ^a^ | 56.04 ± 3.13 | 92.84 ± 4.83 ^***^ | 68.61 ± 8.17 ^##^ |
| Amino acids | Glutamate | Glutamate ^a^ | 1074.95 ± 87.05 | 1194.89 ±77.89 | 1262.67 ± 37.42 |
|  | Aspartate | Aspartate ^a^ | 259.10 ± 26.59 | 214.74 ± 23.62 | 142.94 ± 8.76 ^#^ |
| ***Hippocampus*** | | | | | |
| Amine | Acetylcholine | Choline ^a^ | 56.10 ± 5.06 | 56.51 ± 2.28 | 33.12 ± 1.92 ^###^ |
|  | Serotonin | 5-HIAA ^b^ | 600.37 ± 46.16 | 626.77 ± 37.07 | 458.52 ± 76.93 ^#^ |
|  | Catecholamines | Octopamine ^a^ | 719.12 ± 45.97 | 715.31 ± 51.12 | 582.95 ± 32.86 |
|  |  | 3-MT ^b^ | 8.10 ± 1.49 | 3.80 ± 1.08 ^*^ | 4.00 ± 1.78 |
|  |  | DOPAC ^b^ | 35.60 ± 6.77 | 34.27 ± 10.22 | 32.68 ± 10.29 |
|  |  | HVA ^b^ | 3624.63 ± 913.54 | 4138.56 ± 1159.50 | 2765.44 ± 663.56 |
|  | Polyamines | Putrescine ^b^ | 215.37 ± 22.07 | 628.27 ± 76.87 ^***^ | 332.64 ± 53.05 ^##^ |
|  |  | Spermidine ^a^ | 47.14 ± 3.46 | 54.15 ± 2.18 | 42.60 ± 1.93 ^#^ |
|  |  | Spermine ^a^ | 52.33 ± 1.71 | 69.35 ± 2.23 ^***^ | 55.32 ± 3.35 ^##^ |
|  | Glutamate | Glutamine ^a^ | 262.75 ± 28.35 | 314.42 ± 13.86 | 276.02 ± 25.29 |
|  | Aspartate | Aspartate ^a^ | 141.46 ± 14.77 | 140.10 ± 4.83 | 78.95 ± 5.43 ^##^ |
| Symbols: ^a^, μg/g tissue; ^b^, ng/g tissue, ^*^ *p* < 0.05, and ^**^ *p* < 0.01 indicates significant differences compared with the standard-housing WT mice, ^#^ *p* < 0.05, ^##^ *p* < 0.01, and ^###^ *p* < 0.001 indicates significant differences compared with the standard-housing 5XFAD mice.  Abbreviation: WT, wild type; SH, standard housing; EE, environmental enrichment; 5-HIAA, 5-Hydroxy indoleacetic acid, 3-MT, 3-methoxytyramine; DOPAC, 3,4-dihydroxyphenylacetic acid; HVA, homovanillic acid. | | | | | |
